# Overcoming the Limitations of Forward Osmosis and Membrane Distillation in Sustainable Hybrid Processes Managing the Water–Energy Nexus

**DOI:** 10.3390/membranes15060162

**Published:** 2025-05-26

**Authors:** Muhammad Suleman, Basel Al-Rudainy, Frank Lipnizki

**Affiliations:** Division of Chemical Engineering, Department of Process and Life Science Engineering, Lund University, P.O. Box 124, 221 00 Lund, Sweden; basel.al-rudainy@ple.lth.se

**Keywords:** draw solution, forward osmosis, hybrid process, membrane distillation

## Abstract

Energy-efficient and cost-effective water desalination systems can significantly replenish freshwater reserves without further stressing limited energy resources. Currently, the majority of the desalination systems are operated by non-renewable energy sources such as fossil fuel power plants. The viability of any desalination process depends primarily on the type and amount of energy it utilizes and on the product recovery. In recent years, membrane distillation (MD) and forward osmosis (FO) have drawn the attention of the scientific community because of FO’s low energy demand and the potential of MD operation with low-grade heat or a renewable source like geothermal, wind, or solar energy. Despite the numerous potential advantages of MD and FO, there are still some limitations that negatively affect their performance associated with the water–energy nexus. This critical review focuses on the hybrid forward osmosis–membrane distillation (FO-MD) processes, emphasizing energy demand and product quality. It starts with exploring the limitations of MD and FO as standalone processes and their performance. Based on this, the importance of combining these technologies into an FO-MD hybrid process and the resulting strengths of it will be demonstrated. The promising applications of this hybrid process and their advantages will be also explored. Furthermore, the performance of FO-MD processes will be compared with other hybrid processes like FO–nanofiltration (FO-NF) and FO–reverse osmosis (FO-RO). It will be outlined how the FO-MD hybrid process could outperform other hybrid processes when utilizing a low-grade heat source. In conclusion, it will be shown that the FO-MD hybrid process can offer a sustainable solution to address water scarcity and efficiently manage the water–energy nexus.

## 1. Introduction

The earth’s surface comprises nearly 70% of water, an abundant natural resource, out of which, however, only 3% is drinkable freshwater. The remaining 97% is in the oceans and is non-drinkable due to its high salinity [1,2]. The growth in the world’s population has stressed natural water resources, resulting in global freshwater scarcity, which has become a focused research area within the scientific community. It is expected that by 2050, nearly 2.7–3.2 billion people might be living in water-stressed regions [3]. Desalination technologies offer a solution to this freshwater shortage by converting brackish water and seawater into drinkable water. More than half of the global desalination plants use the reverse osmosis (RO) process due to its simplicity and lower energy cost in comparison with thermal based distillation processes, as shown in Table 1 [4]. Nanofiltration (NF) and Electrodialysis (ED) are other membrane-based processes suitable for desalination. Moreover, several thermal techniques, including multi-stage flash distillation (MSF) and multi-effect distillation (MED), have been developed over the past few decades for seawater desalination. However, these thermal techniques involve high maintenance and operating costs, making them impractical when using low-grade heat sources [5,6]. Alternatives to these conventional methods are MD and FO, which have recently emerged [7,8].

MD is a thermal-driven purification method that relies on a porous hydrophobic membrane. The driving force to generate a flux is the vapor pressure gradient across the membrane. This is achieved by keeping the permeate and feed side at two separate temperatures. The MD process exhibits many attractive features, especially when combined with a low-grade thermal source. These appealing features of MD are its low operating pressures relative to pressure-dependent processes such as NF or RO, producing ultra-pure water due to theoretically 100% rejection in the case of non-volatile components, no need for comprehensive pretreatment, lower energy utilization when waste heat is available, and a higher capability to use low-grade heat sources [7,9,10,11,12,13]. Besides brackish or seawater desalination, MD can be used in many other applications, such as mineral recovery [14], juice and coffee concentrations [15,16,17], liquid desiccant regeneration [18], concentration of carbonate solutions [19], aroma compounds’ recovery [20] removal of volatile organic compounds from wastewater [21], etc.

In FO, a hydrophilic membrane is used, where the key factor is the osmotic gradient. A solution with a high osmotic pressure, referred to as the “draw solution (DS)”, is used on the permeate side of membrane. The osmotic pressure difference generates flux from the feed solution (FS) towards the DS, thereby separating the solutes. FO offers many appealing features, like low energy utilization, less fouling tendency, reduced pretreatment of FS, and minimized need for membrane cleaning, which in turn extends the membrane life cycle [22,23,24].

Despite several potential features of FO and MD, there exist some limitations for each of the processes when implemented separately. The key limitation for FO is the need for regeneration of the DS. When water moves from the feed towards the draw side, the DS becomes diluted. The diluted DS cannot be directly re-used; it requires a regeneration process as further treatment to separate the water from the DS and reconcentrate the DS to maintain a continuous process. Depending on the regeneration process used, regenerating the diluted DS can be more energy-demanding than using a standalone pressure-driven RO process [25]. FO is most suitable when the diluted DS is the end-product, or when the DS could be discarded after the FO operation. Efficient DS recovery with a low energy demand and operation costs is vital for the overall efficiency of the FO process.

Several investigations have shown that FO-RO processes, where RO is employed to regenerate the DS, consume more energy than standalone RO [26,27]. Consequently, FO-RO hybrid processes may be well-suited for desalinating high-salinity seawater and producing ultra-pure drinking water because of the double barrier formed by the membranes. In addition, hybrid FO-RO processes offer efficient treatment for wastewater with a high fouling propensity, thereby reducing the fouling risk for the RO membranes [28].

The major hurdles hindering MD performance are membrane fouling/scaling and high thermal energy demand. Fouling and scaling in MD can take the form of crystallization, biological growth, or particulates. Fouling directly correlates with MD performance by reducing the MD capacity, i.e., the flux across the membrane [29,30,31,32,33]. Additionally, the high operational cost associated with thermal energy consumption in MD can be mitigated by utilizing renewable energy sources or a low-grade heat source.

Until now, researchers have primarily focused on hybrid processes such as FO-NF or FO-RO which are comparatively more energy intensive than standalone RO. While reviews are available on other hybrid processes, such as FO-RO [34,35], there is a lack of reviews on FO-MD hybrid processes. This state-of-the-art review article aims to discuss the robustness and energy efficiency of the FO-MD hybrid process. Firstly, the limitations and inherent characteristics of the standalone MD and FO processes will be briefly evaluated. Next, these inherent characteristics will be combined in a hybrid process which could outperform other hybrid processes, given a low-grade heat source is utilized for the MD and a suitable DS is selected for the FO. Then, the existing literature comparing the efficiency of this technology with other hybrid processes in relation to the water–energy nexus will be reviewed. Finally, key advancements and future prospects will be highlighted to establish this process as an energy-efficient and low-cost desalination solution.

**Table 1:** Energy consumption in different desalination technologies.

Desalination Technology	Plant Capacity (m^3^/Day)	Energy Consumption (kWh/m^3^)	Ref.
RO	100,000–305,000	2.5–4.0	[36]
	NA	2.58–8.5	[37]
	128,000	4–6	[38]
MSF	50,000–70,000	19.58–27.25	[38]
MED	5000–15,000	14.45–21.35	[38]
Mechanical vapor compression	100–3000	7–12	[38]
Thermal vapor compression	10,000–30,000	16.26	[38]
NF	NA	2.54–4.2	[39]
FO (standalone)	NA	0.084–0.275	[40]
	NA	0.11	[22]
MD	1–15	1.58–2.63	[41]

## 2. Forward Osmosis (FO)

### 2.1. Overview

**Figure 1:**
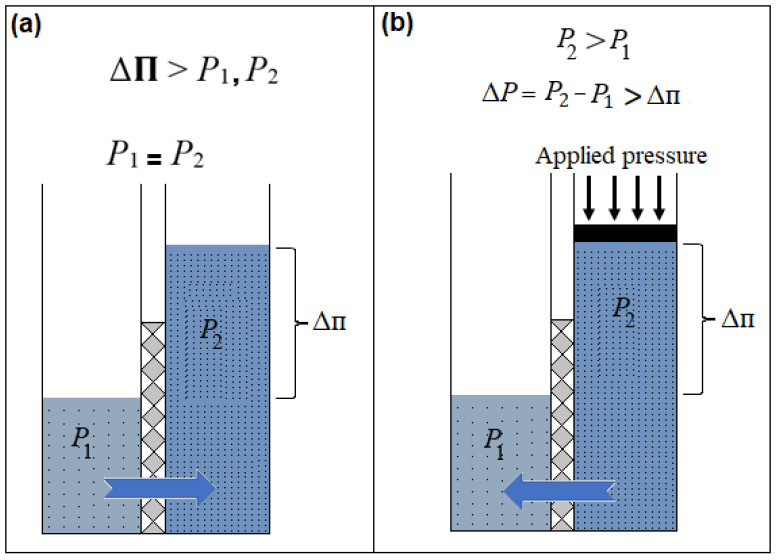
Illustration of (**a**) FO, where *P*_1_ and *P*_2_ represent the hydraulic pressures of dilute and concentrated solutions, respectively, and Δп is the osmotic pressure difference; (**b**) RO, with *P*_1_ and *P*_2_ denoting the hydraulic pressures of dilute and concentrated solutions, respectively, Δп indicating the osmotic gradient, and Δ*P* representing the hydraulic pressure gradient. Figure adapted from [44].

Osmosis refers to the movement of water molecules via a selective membrane from a region having a lower solute concentration to a higher concentration. The membrane rejects the solute, allowing just water molecules to pass through until osmotic equilibrium is achieved. In FO, the difference in solute concentration creates an osmotic gradient that drives the transport of solvent molecules. Compared with RO, FO has shown significantly lower flux for the same membrane area [42,43]. Figure 1 illustrates the differences between FO and RO.

Osmotic pressure is the lowest pressure needed to avoid the osmotic movement of solvent molecules across a membrane. Both experimental and theoretical methods can be applied to measure osmotic pressure. Applying Van’t Hoff’s equation, osmotic pressure is computed as follows:

(1) Π=nMRT

Here, ‘*n*’ represents the Van’t Hoff factor, ‘*M*’ denotes the molarity, and ‘*T*’ and ‘*R*’ stand for the temperature and gas constant, respectively. When using Van’t Hoff’s equation, it is essential for the solute concentration to be sufficiently low so that the solution can be treated as an ideal one.

### 2.2. FO Opportunities and Challenges

The FO process has several applications, including wastewater treatment, water reclamation, desalination, liquid food concentration, and pharmaceutical applications [45]. Some of these applications have been commercialized, including desalination and water reclamation [46]. FO offers many benefits including low energy consumption, low temperature and pressure requirements, low fouling tendency, and high fouling reversibility [47].

The lower energy demand of the FO process, as highlighted in Table 1, positions it as a competitive alternative to conventional desalination processes. Iskander et al. [40] reported a maximum energy consumption of 0.275 kWh/m^3^ for treatment of landfill leachate via the FO process. Similarly, Mazlan et al. [22] reported 0.11 kWh/m^3^ of energy consumption for the FO desalination process. In addition, no hydraulic pressure is required in the FO process, which contributes to its low energy requirements and enhances the reversibility of membrane fouling. Studies have shown that more than 90% of the flux recovery can be achieved by simply backwashing the FO membranes with tap water [48,49]. Further, it has also been claimed that the low fouling tendency in FO is because of the lower fluxes in the FO process [50]. Regardless of these potential benefits of FO, the technology faces several challenges including regeneration of the DS, FO membrane development, concentration polarization (CP), and reverse solute flux (RSF) [51].

In the context of RSF, Hancock et al. [52] studied the effect of different operating conditions on RSF and found that RSF ranged between 80 and 3000 mg/L of produced water for commercially available FO membranes. Conventional strategies to address RSF includes the development of high-selective FO membranes or the use of low-diffusion draw solutes such as responsive DS [53]. Compared to inorganic salts, all responsive DSs have a low RSF. According to Zhang et al. [54], the majority of the responsive DSs have a RSF of 0–0.5 gMH, with pH-responsive DSs showing a particularly low RSF of 0–0.2 gMH. Han et al. [55] produced flat-sheet thin film composite (TFC)-FO membranes using the wet phase inversion method. To enhance the separation performance, they modified the polysulfone substrate with a polydopamine coating. In their experiments, a 2 M NaCl solution was used as the DS, and the resulting RSF was reported as 1.8 gMH. The specific RSF was remarkably low, measured at just 0.075 g/L, highlighting that the membrane fabrication approach is promising to reduce RSF. In another study [56], the support layer was formed by blending polyacrylonitrile and cellulose acetate (CA) via electrospinning and the resulting specific RSF was 0.04 g/L, approximately 90% less than that of standard commercial FO membranes. Thus, novel fabrication techniques for FO membranes play a crucial role in mitigating RSF.

### 2.3. Draw Solution

A major challenge in FO is the careful selection and regeneration of an appropriate DS. The effectiveness of the FO depends on choosing the optimal DS. Over time, the DS becomes diluted, necessitating periodic reconcentration. In cases where the final product is the diluted DS, or when the DS can be discarded after a single operation cycle without needing replenishment, standalone FO may present a potential solution. Therefore, FO is particularly suitable for applications such as wastewater treatment and product concentration [57,58]. Chekli et al. [59] investigated the potential of a fertilizer as the DS for treating a wastewater feed stream. In this case, the diluted DS was suitable for agriculture purposes. Among the nine different tested fertilizers, ammonium phosphate monobasic, ammonium sulfate, and mono-potassium phosphate were selected for long-term use as DSs. The ammonium sulfate DS showed the highest water recovery of 76.2% after a 4-day operation. Similarly, Raval et al. [60] demonstrated the use of fertilizer as a DS for brackish water treatment. Ammonium sulfate, magnesium nitrate, and potassium chloride, each with a concentration of 2 M, were used as the DS. The proposed FO process eliminated the regeneration step, allowing the diluted DS to be directly utilized for agricultural fertigation.

#### 2.3.1. Characteristics of an Ideal Draw Solution

To select a suitable DS, several key factors must be considered. The primary criterion is high osmotic pressure, since the net FO flux is directly proportional to the osmotic gradient. According to Van’t Hoff’s equation (Equation (1)), the osmotic pressure for an ideal solution depends on the Van’t Hoff’s factor and the molar concentration, ‘*M*’. The nature of the solute does not affect the gas constant and temperature. The Van’t Hoff’s factor represents the ratio between the actual and theoretical solute particle concentrations, and both the molar concentration and Van’t Hoff’s factor are dependent on the solute’s solubility. Therefore, an optimal DS requires a high solute solubility to achieve higher osmotic pressures. NaCl or MgCl_2_ are commonly used DSs due to their substantial water solubility, which leads to high osmotic pressures [25,44,61,62,63]. The high diffusion constants of NaCl and MgCl_2_ compared to other alternative draw solutes, such as polymers or nanoparticles, further enhance their suitability as effective options. Additionally, the wide availability of NaCl and its compatibility with various FO membrane materials contribute to its prevalent usage as a DS.

In addition to high solubility and osmotic pressure, an ideal DS should have low viscosity for a wide range of concentrations to facilitate fluid flow. It should also possess a low molecular weight and a high diffusion coefficient. Increased viscosity and molecular weight lead to reduced diffusion coefficients, which can increase internal concentration polarization (ICP) at the DS side of the membrane. To increase FO flux across the membrane and minimize mass transfer resistances, a DS with lower viscosity and molecular weight is essential [25].

Moreover, an optimal DS should ideally exhibit zero RSF. RSF exists as a unique phenomenon in the FO process and presents a major hurdle in developing high-performance FO processes [24,62,64,65,66,67,68,69]. RSF occurs when the DS concentration significantly exceeds that of the FS, causing solute molecules to diffuse back toward the feed side. The negative impacts of RSF on FO performance include a reduced net solution flow from FS towards the draw side, gradual depletion of the DS, increased CP, membrane fouling, and higher operational costs due to the demand for periodic replenishment of DS [64,70]. RSF is, in particular, problematic when the concentrated FS is the desired product, as solutes can contaminate it. Therefore, addressing RSF is necessary for an efficient FO process.

#### 2.3.2. Selection of an Appropriate DS

Choosing an appropriate DS involves several important considerations. The DS must be easily and cost-effectively regenerated. To maintain the necessary osmotic gradient across the membrane during long-term operation, periodic reconcentration of the DS is often essential. Furthermore, producing freshwater with FO generally requires a hybrid process that integrates typical FO with additional membrane-based processes such as MD, NF, RO, ED, ultrafiltration (UF), or distillation processes including MED or MSF.

**Figure 2:**
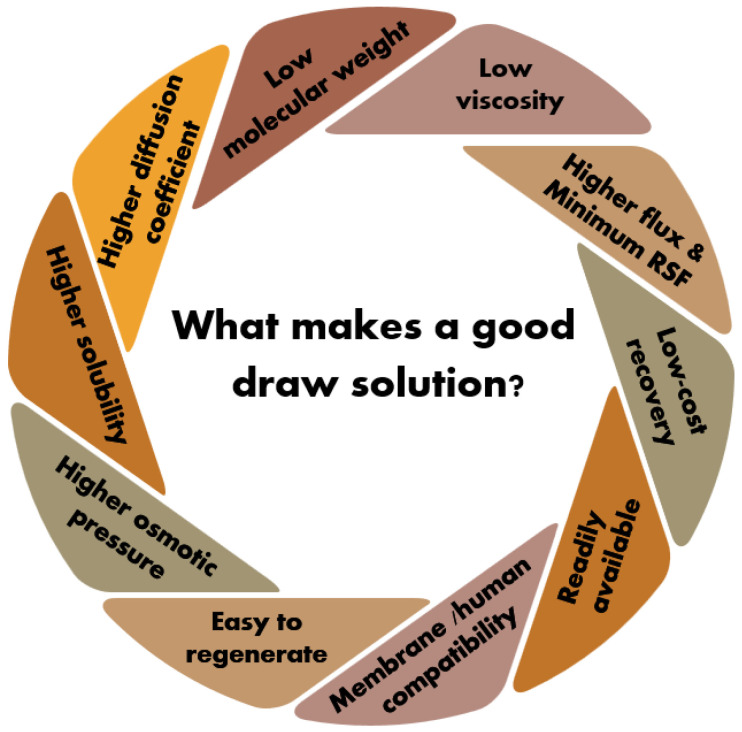
Key characteristics of a draw solution.

In all these hybrid concepts, FO is the initial process step to extract water into the DS, followed by a secondary step to generate water and to regenerate the DS, ensuring the overall feasibility of the FO process [71,72,73,74,75,76,77,78,79,80]. These supplementary procedures contribute a considerable part of the overall energy consumption, potentially increasing both the energy requirements and operational expenses [81]. In fact, integrated FO-RO processes are reported to consume more energy than a standalone RO unit for desalination [82]. However, an economic analysis of an FO-low pressure RO hybrid process for seawater desalination revealed 16% lower water production costs compared to standalone seawater RO [83]. Thus, the selection of an appropriate DS and its respective regeneration step is critical to make FO hybrid processes economically viable. Current DSs either involve expensive solutes or incur high regeneration costs [25,84]. Choosing as appropriate DS extends from commonly accessible and cost-effective inorganic draw solutes [85], to innovative stimuli-responsive options [54], such as thermo-responsive polymers [86], highly charged ionic substances [87], and hydrogels [88]. Low-temperature heat sources can be employed to regenerate the stimuli-responsive DSs, thereby lowering the associated regeneration costs [86]. However, a significant limitation of these draw solutes is the high cost of synthesis [25,84]. As a result, none of the currently available draw solutes can be considered perfect for the FO process [25]. Therefore, achieving a sustainable hybrid process requires an energy-efficient and economically viable approach to DS regeneration. The key attributes of an ideal DS are outlined in Figure 2.

#### 2.3.3. Classification of Draw Solutes

Draw solutes can be categorized based on their inherent properties or the techniques used for their regeneration. The existing literature features a diverse array of draw solutes, chosen according to the specific demands of the process and the methods used for their recovery.

##### Inorganic Salts

Inorganic salts are often used as DSs due to their solubility in water, which facilitates higher osmotic pressures and elevated fluxes [89]. Among these salts, NaCl is the most commonly used DS due to its high solubility, low viscosity at higher concentrations, and non-toxic nature [85]. Additionally, NaCl is widely available, cost-effective, and could be easily reconcentrated through thermal-driven membrane processes such as MD or pressure-driven membrane processes like RO. These attributes make NaCl an attractive choice as a draw solute.

Table 2 provides an overview of some inorganic salts that have been employed as DSs for FO, highlighting that NaCl and MgCl_2_ are the most widely used inorganic draw solutes in the past years. A key advantage of using inorganic draw solutes is their easy recovery by thermal-driven membrane processes like MD. However, monovalent salts, such as NaCl, KCl, KNO_3_, KBr, and NH_4_Cl, tend to have higher RSFs because of their smaller hydrated radii and lower charge density compared to divalent or trivalent inorganic salts [89,90]. To address this issue, divalent inorganic salts like CaCl_2_ and MgCl_2_ have been proposed as alternatives [89,90,91,92]. A study by Eddouibi et al. [90] evaluated the RSF performance of NaCl and MgCl_2_ and revealed that MgCl_2_ significantly outperformed NaCl, showing a 28.87% lower specific RSF ratio, regardless of the solute concentration.

**Table 2:** Inorganic salts and fertilizers used as DSs for FO along with their respective regeneration techniques (where applicable).

DS	DS Conc.	FS	FO Membrane	Flux (LMH)	Recovery	Ref.
NaCl	1 M	DI water	Flat-sheet CTA membrane from FTSH20©, Albany, OR, USA	NA	NA	[90]
MgCl_2_	1 M	DI water	CTA flat-sheet membrane from FTSH20©, USA	NA	NA	[90]
NaCl	0.5 M	DI water, wastewater	CTA membrane from HTI, Albany, OR, USA	6	MD	[93]
NaCl	0.8 M	Wastewater	CTA based FO membrane from HTI, USA	9–52	NF	[94]
NaCl	1.5 M	Raw sewage	Flat-sheet cellulose-based membrane from HTI, USA	8	MD	[95]
MgCl_2_	1.5 M	Digested sludge	Flat-sheet CTA membrane from HTI, USA	9	MD	[91]
NaCl	4.82 M	Waste landfill leachate	TFC membrane from HTI, USA	NA	MD	[96]
NaCl	0.5–2 M	BSA solution	Hydrophobic PBI NF hollow fiber membrane	NA	MD	[72]
KCl	2 M	DI water	CA membrane from HTI, USA	22.6	NA	[97]
NaNO_3_				20.35		
KNO_3_				15.8		
NH_4_NO_3_				14.9		
NH_4_Cl				19.07		
(NH_4_)_2_SO_4_				19.23		
NH_4_H_2_PO_4_				15.51		
Ca(NO_3_)_2_				17.91		
(NH_4_)_2_HPO_4_				13.88		
CaCl_2_	1.6 M	DI water	PA based flat-sheet TFC membrane from HTI, USA	NA	MD	[92]
HCOONa	4.1 M					
KBr	3.2 M					
LiBr	2.2 M					
LiCl	2.6 M					
MgCl_2_	1.5 M					
Na(C_2_H_5_COO)	4.1 M					
NaCl	3.0 M					

PBI—polybenzimidazole; PA—polyamide.

##### Organic Compounds

Along with inorganic salts, organic compounds have also been considered as DSs for FO. Organic compounds potentially offer significant advantages, including higher fluxes and lower RSF due to their relatively higher molecular weight compared to the simpler inorganic compounds [98]. Ge et al. [99] studied the use of polyacrylic acid sodium (PAA-Na) as an polyelectrolyte draw solute for the dehydration of wastewater with FO, followed by MD for DS regeneration. They identified a draw solute concentration of 0.48 g/L and temperature of 66 °C as the most effective conditions for wastewater reclamation. Besides MD, PAA-Na can be regenerated using pressure-driven membrane processes like UF [100]. Similarly, research has been conducted on 2-methylimidazole-based organic compounds, including both the charged and neutral draw solutes, with DS regeneration achieved through the integration of MD [79]. A selection of organic compounds used as draw solutes is presented in Table 3 [100,101,102,103,104,105].

The organic compounds listed in Table 3 offer the advantage of their large molecular size, resulting in significantly reduced RSF values, positioning them as potential alternatives to conventional inorganic salts. For instance, Jun et al. [102] compared the specific RSF of polyethyleneimine (PEI) with those of NaCl and MgSO_4_. At a solute concentration of 2000 ppm, the specific RSF values for NaCl, MgSO_4_, and PEI were found to be 640, 2.9, and 2.4 mg/L, respectively. The higher molecular weights of the organic compounds, compared to inorganic salts like NaCl, facilitated their regeneration using membrane processes [101]. However, an increase in molecular weight can also result in more viscous DSs, which impacts their ability to circulate, increases their CP, and ultimately affects the FO performance [105].

##### Thermo-Responsive Polymers

Thermo-responsive polymers represent a special class of organic compounds. To enhance the regeneration process, polymers exhibiting thermo-responsive behavior in aqueous solutions have been considered as draw solutes [106,107]. These polymers feature a lower critical solution temperature (LCST), which regulates their solubility in water. Below the LCST, they form a homogenous single-phase aqueous solution, but above this threshold temperature, they undergo a phase separation and become insoluble in water [98]. These polymers can be then concentrated by density-driven methods such as decanters and coalescers.

Zhao et al. [106] employed a poly(sodium styrene-4-sulfonate-co-n-isopropylacrylamide) (PSSS-PNIPAM) copolymer as a DS. During the preparation of the copolymer, the concentration of PSSS was adjusted between 5 and 20 wt.% while the concentrations of PNIPAM were varied between 19 and 16 wt.%. Remarkably, at a PSSS concentration of 15 wt.%, RSF and water flux values of 2 gMH and 4 LMH, respectively, were attained. This represents a substantially lower RSF value compared to the 90 gMH achieved with the same membrane when NaCl was used as a draw solute. The larger size and expanded structure of PSSS-PNIPAM, in contrast to inorganic salts, contribute to this decreased RSF value [106]. Due to their temperature-responsive characteristics, these polymers can be efficiently reconcentrated using MD. The selection of an appropriate polymer as a DS primarily depends on its LCST, membrane material compatibility, solubility in water, and ease of phase separation above its LCST, resulting in distinct polymer-rich and water-rich phases.

##### Magnetic Nanoparticles (MNPs)

Although most of the inorganic compounds demonstrate high osmotic pressure, the problem lies in regeneration of these DSs requiring a secondary process. To overcome this, MNPs coated with hydrophilic polymers and dispersed in water have been proposed as a DS. The coating is necessary to prevent agglomeration of the NPs. After the DS dilution, these coated MNPs can be recycled under the influence of an external magnetic field [108]. Magnetite Fe_3_O_4_ is the most commonly synthesized MNP used in the FO process for this category [106,109,110]. Dey et al. [111] synthesized Poly sodium acrylate (PSA)-coated MNPs for water desalination by FO. The PSA-MNP synthesized DS achieved an FO water flux of 5.32 LMH, which is approximately two to three times higher than the flux observed with the PSA solution alone. Further, Ling et al. [112] developed a DS by coating MNPs with triethylene glycol for use in the FO process. These coated MNPs demonstrated efficient recovery through the application of a magnetic separator. The combination of high FO flux and the simple cost-effective regeneration of MNP-based DSs positions them as a promising option. The study confirmed nearly complete recovery, with 100% of the dispersed Fe_3_O_4_ MNPs successfully reclaimed within 7 min by applying a magnetic field [113]. 

**Table 3:** Overview of organic compounds, thermo-responsive polymers, MNPs, and high charge ionic compounds used for FO with their respective regeneration methods.

DS Category	DS	FO Membrane	Flux (LMH)	Recovery	Ref.
Organic compounds	PAA-Na	Flat-sheet CTA membrane from HTI (USA)	5	UF	[100]
	Na salt of Poly (aspartic acid)	TFC FO membrane from HTI (USA)	31.8	NF, MD	[101]
	PEI	TFC membrane from HTI, USA	NA	NF	[102]
	Dimethyl ether	NA	NA	Thermal heating	[104]
	Sodium Polystyrene sulfonate	Commercial TFC FO membrane from HTI (USA)	13	UF	[105]
Thermo-responsive polymer	PAGB	Commercial flat-sheet CTA membrane (HTI, USA)	NA	NA	[114]
	EO-PO copolymer	Hollow fiber FO membrane (TOYOBO, Osaka, Japan)	NA	Coalescer and NF	[115]
	Pluronic^®^ L-35	TFC hollow fiber FO membrane	1.22	Phase separation	[116]
MNPs	Citrate coated Fe_3_O_4_	Commercial CTA membrane (HTI, USA)	17.3	Magnetic separation	[117]
	PEG diacid coated MNPs	Flat-sheet membrane (HTI, USA)	>10	NA	[118]
	Dextran coated Fe_3_O_4_ MNPs	HTI FO membrane	NA	Magnetic separation	[109]
High charged compound	EDTA sodium salt	CTA FO membrane (HTI, USA)	8.45	NF	[87]
	EDTA-2Na	NA	NA	MD	[119]
Hydrogels	Hydrolyzed polyacrylamide	Polyamide-based TFC FO membrane	NA	NA	[103]
	Polyacrylamide	NA	0.36	Pressure stimuli	[88]

PAA-Na—polyacrylic acid sodium; PAGB—Poly (propylene glycol-ran-ethylene glycol) monobutyl ether; EO-PO—Ethylene oxide-propylene oxide.

### 2.4. DS Regeneration Techniques

**Figure 3:**
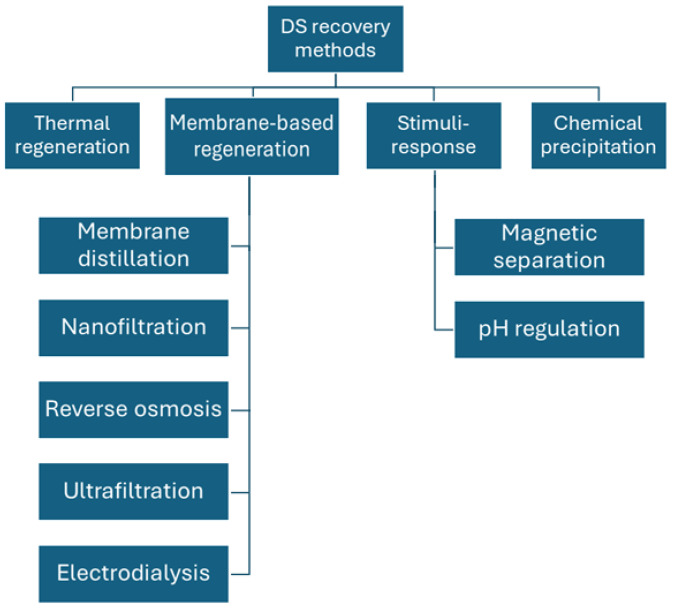
Classification of DS recovery methods [84].

One of the key limitations in FO is the regeneration of the DS, which is essential for a continuous FO process. The type of regeneration process and its associated costs are important in selecting an appropriate DS. Standalone FO may offer a potential solution when the diluted DS is the final product, or when the DS can be discarded after a single operation. For instance, the use of concentrated fertilizer as a DS, where the diluted DS can be used for fertigation, does not require any regeneration process [59]. DS recovery techniques can be broadly categorized into thermal regeneration (e.g., heating at controlled temperatures) [120], membrane-based regeneration (utilizing selective membranes for separation) [74], stimuli-responsive methods (activation by specific triggers, such as pressure or temperature) [118], and chemical precipitation (removal of solutes via chemical reactions) [121]. These methods are visually summarized in Figure 3. Additionally, an overview of their advantages and disadvantages is detailed in Table 4.

**Table 4:** Overview of the DS regeneration techniques.

Category	Recovery Method	DS Type	Advantages/Disadvantages	Ref.
Thermal regeneration	Heating at 60 °C	Switchable polarity solvent	1. Thermal regeneration is energy intensive. 2. Low-grade or renewable heat sources can be used. 3. Product quality is poor.	[120]
	Heating at 60 °C	NH_3_/CO_2_		[122]
	Heating above 60 °C	Thermo-responsive polymer		[86]
Membrane-based regeneration	MD	NaCl	1. Besides MD, other processes are less energy intensive. 2. High water quality and recovery rate.	[123]
	NF	MgCl_2_		[124]
	UF	Polyelectrolyte		[100]
	RO	Real seawater		[125]
	ED	NaCl		[74]
Stimuli response	Magnetic separation	Functionalized MNPs	1. Only suitable for materials whose solubility changes with pH. 2. Low water quality. 3. Cost-effective.	[108]
	Magnetic separation	Fe_3_O_4_ MNPs		[109]
	pH regulation	EDTA-2Na		[126]
Chemical precipitation	Metathesis precipitation	Copper sulfate	1. The product could be toxic. 2. Chemicals are costly.	[121]

## 3. Membrane Distillation

### 3.1. Overview

Distillation processes can be broadly classified as isothermal and non-isothermal processes. MD, a non-isothermal purification process, has emerged in the past two decades as an alternative to conventional processes like MSF and MED [7]. MD is a unique membrane purification process that uses a hydrophobic membrane to separate the feed stream from the permeate side. The transport of vapors is attained by establishing a vapor pressure difference across the membrane.

**Figure 4:**
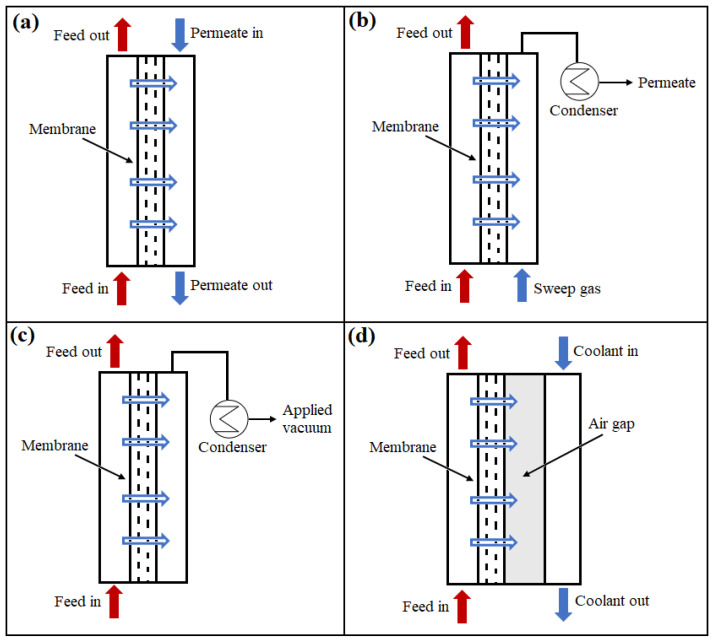
MD configurations: (**a**) DCMD, (**b**) SGMD, (**c**) VMD, and (**d**) AGMD.

Depending on the requirements, MD can be implemented in four different process configurations: vacuum membrane distillation (VMD), direct contact membrane distillation (DCMD), sweeping gas membrane distillation (SGMD), and air gap membrane distillation (AGMD) [33,127,128,129,130,131,132,133,134,135,136,137,138,139]. These configurations differ primarily in how the vapors are collected at the permeate side. Figure 4a–d demonstrates the fundamentals of these four different types of MD arrangements.

### 3.2. Factors Limiting Commercialization and Industrial Application of MD

While MD offers many advantages, such as a high rejection rate, lower operating pressure, and potential for zero liquid discharge, there are still several limitations associated with standalone MD. These limitations hinder its wide use in industrial applications, increase operating costs, and require additional energy input. In the following the key limitations, namely fouling, pore wetting, and heat energy consumption, will be discussed briefly.

#### 3.2.1. Fouling

Fouling in MD means the deposition of unwanted deposits on the membrane surface, leading to a decline in MD performance. Despite significant advancements over the past decade, MD remains susceptible to membrane fouling. The primary types of fouling in MD are biological, particulate, and crystallization fouling [140,141,142]. Particulate fouling occurs due to the accumulation of suspended particles on the membrane surface. For instance, iron oxide has been identified as a common particulate foulant in various MD studies [31]. Research by Gryta [141] highlighted the impact of iron oxide fouling during MD treatment of effluents from a water treatment plant, observing an 8% reduction in MD flux over 20 h of operation. The primary source of iron oxide fouling in MD systems is the corrosion of metal components [143]. Crystallization fouling, or scaling, results from crystal growth and is commonly observed when the feed contains salts [144]. Drioli and Wu [145] conducted MD experiments using a feed containing 0.58 wt.% NaCl and observed a significant flux reduction of 72% within the first 3 days of operation. In practical applications, fouling in MD often involves a combination of different types of fouling [146]. Operating MD processes at temperatures of 70 °C or higher have shown notable reductions in biological fouling [142]. Gryta [147] investigated the impact of temperature on microbial growth during MD, using saline wastewater containing yeast as the feed. At 80 °C, bacteria were detected on the membrane surface; however, when the feed temperature increased to 90 °C, no bacteria were observed. Likewise, in an AGMD study by Meindersma et al. [148], which utilized pond water as feed, biofouling led to a noticeable decline in flux after 800 h of operation.

Fouling in MD varies with FS characteristics, operating conditions, membrane properties, and foulant characteristics [31]. For instance, fouling effects tends to grow with a smaller pore size and higher hydrophobicity of the MD membrane [149]. Fouling and scaling are particularly important because they impact MD performance in several ways. They increase operating costs due to the need for periodic membrane cleaning, reduce capacity, and ultimately deteriorate the membrane, thus shortening the membrane life cycle [32,150,151]. Fouling leads to pore blocking and membrane wetting, resulting in a decline of flux over time. Since fouling and scaling are time-dependent processes, their effects on MD performance cannot be ignored [7].

To mitigate fouling in MD, several strategies can be employed. These include pretreatment of the FS, flushing and chemical membrane cleaning, use of antiscalants, increasing feed flow velocity, or incorporating turbulence promoters [152,153,154,155]. One possible solution to prevent MD fouling in treatment of wastewater with high fouling propensity is to employ an FO-MD hybrid process, where FO operates as a pretreatment process to enhance the fouling resistance of the MD [156]. Membrane fouling is a complex phenomenon affected by several parameters and understanding these factors is the first step towards addressing the issue. Thus, fouling is a significant drawback of MD that, if left unaddressed, can cause a substantial flux decline over time, reduce the membrane life cycle, and increase operational costs.

#### 3.2.2. Membrane Pore Wetting

Pore wetting takes place when liquid penetrates the membrane pores, significantly reducing the permeate quality and thus posing a major hurdle in the commercialization of MD [157]. This time-dependent phenomenon often manifests after long-term MD operation [158]. Once the pore becomes wetted, feed can flow through it and contaminate the permeate [158,159]. Major factors that facilitate pore wetting include high transmembrane pressure, i.e., exceeding the liquid entry pressure (LEP), membrane degradation from prolonged operation, feed containing hydrophobic materials such as oil droplets or scalants such as calcium carbonate, and condensation in the membrane pores due to low temperatures [156,157,159,160,161,162].

**Figure 5:**
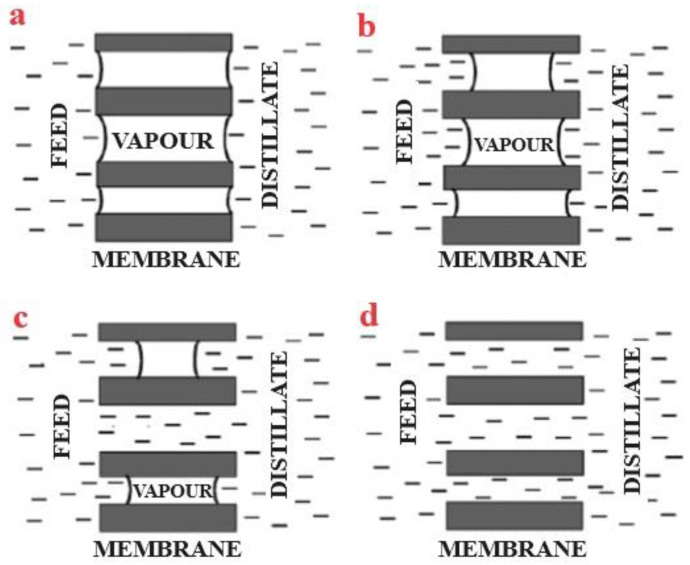
Pore wetting: (**a**) non-wetted, (**b**) surface-wetted, (**c**) partially-wetted, and (**d**) fully-wetted (adapted from [30]).

LEP is a characteristic of hydrophobic membranes and represents the lowest transmembrane pressure needed for the FS to enter the membrane pores [163]. As described by Franken et al. [164], LEP can be evaluated using the following equation:

(2) $$LEP=\frac{-2G_{p}\sigma cos\theta }{r_{max}}$$

where $G_{p}$ represents the pore geometric coefficient, σ denotes the surface tension of the FS, θ represents the contact angle between the FS and membrane surface, and $r_{max}$ is the largest membrane pore size. A higher LEP can be attained by increasing the geometric pore coefficient, surface tension, and contact angle, as well as by using membranes with smaller pore sizes. Subject to the degree of wetting, membrane pore wetting could be categorized into four types, as shown in Figure 5: non-wetted, surface-wetted, partial-wetted, and fully-wetted [30]. A surface-wetted membrane maintains a gap between the permeate and FS, allowing vapors to pass through the membrane. However, in partial-wetted or fully wetted membranes, low-quality permeates are produced as the FS can flow through the membrane.

Gryta et al. [160] studied the pore wetting phenomenon by using NaCl solutions and Baltic seawater, which contains foulants like calcium carbonate, with polypropylene (PP) membranes. After several hours of operation, the PP membrane used with the NaCl solutions did not experience any wetting phenomenon, whereas the same PP membrane used with Baltic seawater was partially wetted. This was explained by the fact that the NaCl solutions are non-foulant feeds, whereas the Baltic seawater contained various foulants that resulted in partial wetting of the membrane. Experimental evidence for pore wetting associated with the condensation of vapors inside the pores can also be found in the literature [164,165]. Additionally, Guillen-Burrieza et al. [165] attributed the pore wetting phenomenon to a sudden drop in the temperature difference across the membrane. Fouling and membrane wetting are interrelated in MD since fouling leads to progressive wettability in the MD, so that the feed can penetrate the pores and contaminate the permeate [7,32].

#### 3.2.3. High Thermal Energy Consumption

The high thermal energy demand in MD limits its industrial scale applications, making it less competitive than other desalination technologies. Given the non-isothermal nature of MD, its thermal efficiency and specific thermal energy consumption (STEC) are key parameters for evaluating MD performance. Several theoretical as well as experimental studies address the energy requirements and thermal efficiency of MD [166,167,168,169,170,171]. Most of the energy is consumed for the heating of the FS to provide the latent heat for evaporation [172]. Guan et al. [173] found that 97.8% of the total energy required for MD crystallization was used for heating of the FS. Sensible heat losses owing to conduction through the membrane material also impact the thermal energy utilization in MD. These losses can be reduced by selecting appropriate materials, such as polymeric membranes with low thermal conductivity. Additionally, suitable membrane characteristics, like membrane pore size, porosity, thickness, membrane tortuosity, etc., can help to mitigate conduction heat losses [174,175,176]. Al-Obaidani et al. [11] assessed the effect of these characteristics on DCMD performance, revealing a 50% reduction in thermal efficiency and a 26% decrease in flux when the membrane’s thermal conductivity increased five-fold from 0.1 to 0.5 W/mK. Similarly, Tlili et al. [177] performed a parametric study to investigate the energy consumption in an MD setup and revealed that increasing the membrane thickness and porosity significantly decreased the energy demand by lowering heat losses from the feed to the distillate side. Additionally, the high energy demand of MD can be reduced by addressing the temperature polarization (TP) phenomenon and implementing multi-staging. The TP issue is more obvious in long MD membrane modules due to the increased heat loss through the membrane, which leads to a reduced distillate flux [178]. This limitation hinders the large-scale implementation of the technology. Dutta et al. [179] analyzed single- and multi-stage DCMD and achieved a 35% decrease in the STEC by switching from single to multi-stage, based on a feed flow rate of 40 LPH. Several researchers have incorporated heat recovery units in MD systems to recycle thermal energy, thereby decreasing overall thermal energy consumption [180,181,182]. Kim et al. [180] achieved a 43% reduction in STEC based on a DCMD setup combined with a heat recovery unit compared to a standalone DCMD system. In addition to conventional methods for improving MD’s thermal efficiency, several novel approaches, such as photothermal and electrothermal heating, have shown great potential [172,183]. In photothermal heating, light-absorbing materials like carbon nanotubes or silicon are incorporated into the MD membranes. Once exposed to light, these light-absorbing materials adsorb light energy and convert it into heat, increasing the membrane surface temperature. This localized heating of the FS reduces the overall thermal energy input required.

In a study performed by Wu et al. [184], membranes coated by carbon black nanoparticles and SiO_2_/Au nano shells revealed a 33% increase in permeate flux in a lab-scale DCMD setup, along with a significant increase in thermal efficiency under simulated sunlight. For electrothermal heating, membranes are coated with a thin layer of conductive material. When electric current passes through the membrane, heat is generated due to resistance, maintaining a higher membrane surface temperature and reducing thermal energy demand. In a similar study by Ahmed et al. [185], PP membranes coated with carbon nanostructures achieved a more than 50% reduction in STEC for feed temperatures of 40, 50, and 60 °C.

Other key sources of energy consumption in MD include vapor condensation and electrical energy consumption for the pumps.

## 4. Integrated FO-MD Process

### 4.1. Fundamentals of the Hybrid Process

The concept of hybrid FO-MD processes is based on the integration of FO with MD in innovative hybrid processes that combine FO as an initial step for water transport from the feed into the DS, while MD acts as a secondary step for DS regeneration [99]. Effective implementation of this hybrid method requires a significant input of thermal energy, as the FS for MD must be sufficiently heated to operate efficiently.

**Figure 6:**
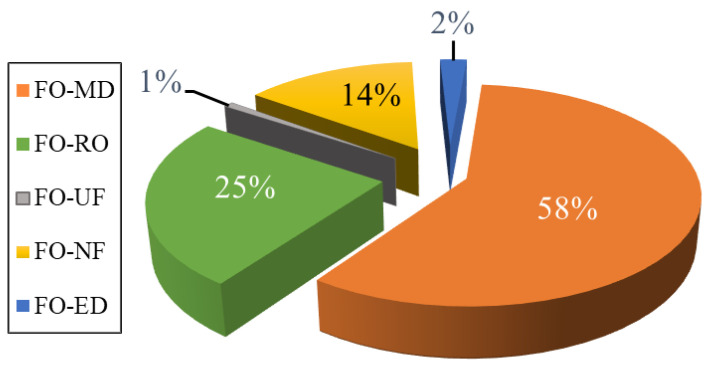
Distribution of regeneration technologies for FO DS, as per Google Scholar data (search conducted on 17 December 2024).

Apart from MD, various other methods have been considered as a secondary step for DS regeneration. These include RO, NF, UF, MSF, MED, and ED [77,94,112,186,187,188]. However, in the literature, MD is the most common regeneration step for the DS. Figure 6 summarizes the distribution of technologies used for DS regeneration in the literature. In over half of the hybrid processes reported, MD is considered as a regeneration process. This preference is due to several factors: MD is far less vulnerable to the salinity and osmotic pressure of the DS in contrast to pressure-driven membrane filtration processes like NF and RO [189]. MD features the highest rejection rates (~99.99%) for non-volatile components among the considered regeneration technologies, thus making it particularly suitable for nonvolatile DSs [190]. MD is effective for regenerating both monovalent and divalent non-volatile ionic draw solutes [191]. Additionally, MD is highly effective in treating hypersaline solutions, making FO-MD an attractive combination, as highly concentrated ionic DSs can be efficiently regenerated using MD. Some of the thermal processes, such as energy-intensive evaporation, can offset the low energy consumption advantage of FO when integrated into a hybrid process.

FO-MD outperforms standalone FO process exclusively when inorganic salts are used as the DS. This is because of the increase in the osmotic pressure of the inorganic DS induced by the high temperature. FO water flux is highly dependent on the osmotic gradient across the membrane; thus, the high temperature assists in an increase in the FO water flux compared to the standalone FO process. Mat Nawi et al. [156] compared the performance of the FO-MD hybrid process to that of the standalone FO process. Wastewater from the petroleum industry and 0.6 M NaCl solution were used as the feed and DS. Their results indicated an increase in the FO water flux from 11.17 to 30.19 LMH, when the temperature of the DS increased from 20 to 60 °C. Thus, coupling MD with FO outperforms the standalone FO process and simultaneously facilitates the regeneration of the DS.

**Figure 7:**
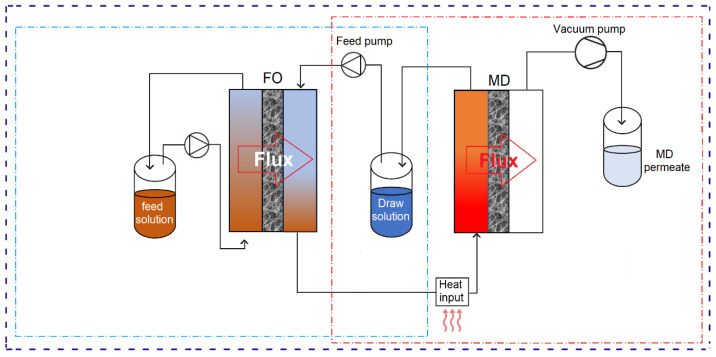
Schematic illustration of the FO-MD hybrid process.

Despite their advantages, FO-MD hybrid processes often face challenges, such as a relatively low DS recovery rate, which is attributed to the limited MD flux. This issue can be addressed by increasing the MD membrane area to enhance performance. The final product of the FO-MD process is condensed vapor on the permeate side. Figure 7 provides a schematic representation of the typical FO-MD hybrid process design, showcasing the integration of both technologies through a shared DS tank. While this design is standard at the laboratory scale, it is often not economical for commercial applications [192]. To reduce the footprint and capital cost for large-scale operations, sealing the FO-MD hybrid system into a single module presents a viable and cost-effective solution.

### 4.2. Opportunities to Integrate FO with MD in a Hybrid Process

As discussed in Section 3.2.3, employing MD as DS regeneration process is thermally less efficient compared to pressure-driven separation technologies or ED [193]. The high thermal demand associated with MD makes the FO-MD hybrid process energy-intensive. However, the potential of MD to harness low-grade heat sources from industries, power stations, or renewable sources like solar or geothermal reservoirs can help to mitigate the high thermal energy costs [46,194,195,196].

Dow et al. [197] demonstrated a pilot-scale DCMD setup that utilized wasted heat at 38 °C from a gas-fired power plant to treat wastewater. The STEC was measured to be 1500 kWh/m^3^, and the plant capacity could reach up to 8000 m^3^/day of desalinated water on the heat available. Similarly, Xu et al. [198] used waste heat from a ship engine to operate a pilot VMD plant for on-board seawater desalination, achieving a rejection rate of 99.99% at a 55 °C feed temperature. Besides industrial waste heat, geothermal or solar heat sources can also be utilized to provide heat for MD. Koschikowski et al. [199] developed eight solar-based MD systems with capacities ranging from 100 to 500 L/day of treated water, as well as larger pilots with capacities up to 10 m^3^/day of treated water. These MD plants were entirely self-sustained, relying solely on solar energy. Sarbatly et al. [200] evaluated geothermal energy consumption in a cross-flow VMD system and found that feeding warm geothermal water directly into the unit saved nearly 95% of overall energy consumption compared to using distilled water. In a related economic survey of a 20,000 m^3^/day of treated water plant, a water production cost of USD 0.5/m^3^ was reported when the facility operated with geothermal energy and USD 1.22/m^3^ without it. These findings highlight how the FO-MD hybrid process can capitalize on low-grade or renewable heat sources to achieve significant energy cost savings.

**Figure 8:**
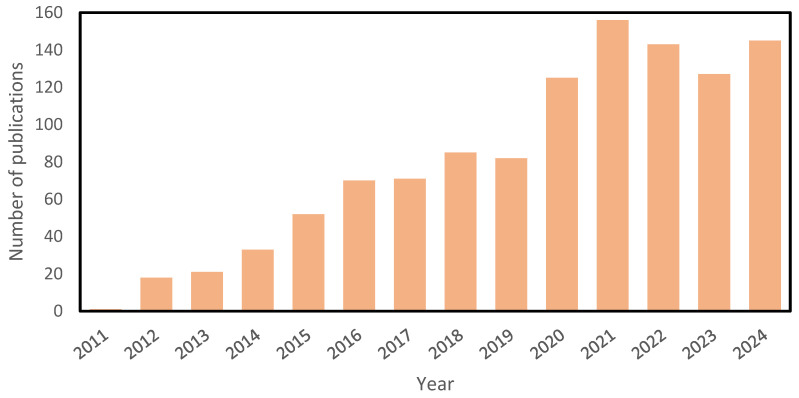
The research trajectory of FO-MD hybrid processes from 2011 to 2024, as per Google Scholar data, using the search term “FO-MD hybrid processes”. This search was conducted on 17 December 2024.

Thus, the integration of FO with MD has gained significant attention in recent years, as illustrated in Figure 8. The hybrid approach has the potential to address the limitations of each individual technology while enabling the production of ultra-pure water with distillate quality below 10 µS·cm^−1^ [201]. The upstream FO process effectively removes a substantial portion of volatile contaminants and foulants, ensuring that the downstream MD process operates with reduced risks of fouling and pore wetting [156,202,203].

Husnain et al. [202] studied an FO-MD hybrid process for the extraction of COD and ammonium from ammonium chloride solution and non-fat dry milk, respectively. Non-fat dry milk with an initial COD of 985 mg/L as the FS and 1 M NaCl as the DS were adopted. After 48 h of operation, the COD content in the FO FS reached 5043 mg/L. The COD concentration in the DS revealed that the FO process alone could achieve 90% of the COD removal before MD. This demonstrates how an upstream FO process eliminates organic contaminants, eventually addressing pore wetting and fouling challenges in the downstream system. In the case of FS containing 0.3–0.495 g/L ammonium chloride, only 0.18 mg/L of ammonium was found in the MD permeate, representing about 100% removal of ammonium.

Similarly, Xie at al. [95] used an FO-MD hybrid process for raw sewage treatment with 1.5 M NaCl as the DS, achieving total organic carbon (TOC) removal rates from 91 to 98%. In this scenario, standalone MD would have been vulnerable to pore wetting caused by organic contaminants.

**Figure 9:**
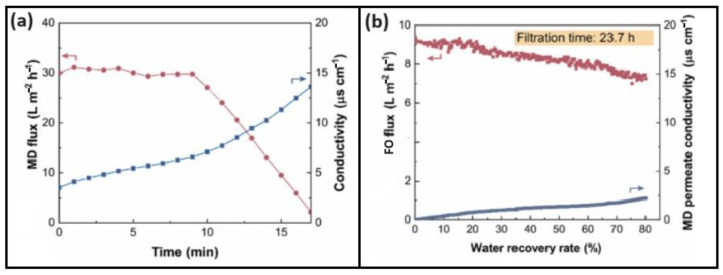
(**a**) Fluxes and conductivity of the standalone MD for textile wastewater treatment (feed temperature: 55 °C, permeate temperature: 10 °C); (**b**) FO fluxes and MD permeate conductivity of the FO-MD hybrid process (FO feed temperature: 22 °C, DS temperature: 35 °C, MD permeate temperature: 8 °C). FO membrane: graphene oxide. The FO membrane was fabricated via interfacial polymerization. MD membrane: Commercial PTFE with a pore size of 0.45 µm from Chanqi, China [206].

Treating textile wastewater directly through MD also includes significant challenges, as indicated by several studies [163,204,205]. This is mostly due to the pore wetting of the membrane caused by organic foulants, and surfactants present in the textile wastewater [163]. Wu et al. [206] treated real textile wastewater using both standalone MD and an FO-MD hybrid process with aqueous NaCl as DS. Their findings revealed that the FO-MD process is more effective, as it overcomes the wetting issues with the MD membrane observed in the standalone MD process. Using standalone MD, a significant decrease in flux was observed after 11 min, having a sharp increase in the permeate conductivity from 3.5 to 13.7 µS·cm^−1^ (Figure 9a). In contrast, the effectiveness of the FO-MD showed that the conductivity of the MD permeate was still 3.9 µS·cm^−1^ after 23.7 h of filtration (Figure 9b). This difference in process output could be attributed to the fact that, in standalone MD, the membrane quickly lost its hydrophobicity, leading to a reduction in rejection efficiency. In the case of the FO-MD hybrid process, the surfactants in the textile wastewater were removed by the initial FO step, ensuring that the MD membrane was less susceptible to wetting.

**Figure 10:**
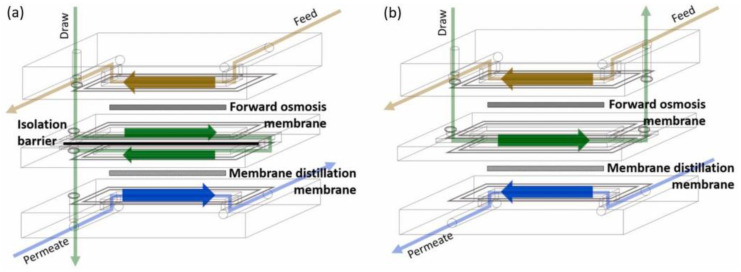
Illustration of the integrated FO-MD unit (**a**) with isolation barrier, and (**b**) without isolation barrier [203].

Common FO-MD hybrid processes consist of separate FO and MD modules. However, such an integration of both the processes can lead to a higher capital cost and footprint compared to a hybrid FO-MD sealed in one module [207]. Accordingly, the direct integration of FO with MD in one module was initially patented by Cath et el. [208], as illustrated in Figure 10b. Afterwards, the direct integration of FO with MD in one module with an isolation barrier was patented by Ghaffour et al. [209], and investigated by Kim et al. [207]. The novel FO-MD module incorporated an isolation barrier to minimize overall energy consumption and enhanced the osmotic potential of the DS. This module featured two distinct flow channels, effectively separating the FO permeate from the MD FS using an isolation barrier, as illustrated in Figure 10a. This overcomes a limitation of the initial design provided by Cath et al. [208], where heat from the MD feed could transfer to the FO permeate, reducing heat availability and subsequently impacting MD flux [203,207]. In contrast, the proposed module allows the MD feed to fully benefit from the maximum available temperature, reducing the need for thermal energy. Additionally, the isolation barrier improves hydrodynamics, creating turbulences that reduce CP, mitigating fouling/scaling, and thus enhancing the efficiency of both processes [210]. Therefore, compact FO-MD modules with isolation barriers are offering the best choice for FO-MD hybrid processes.

**Figure 11:**
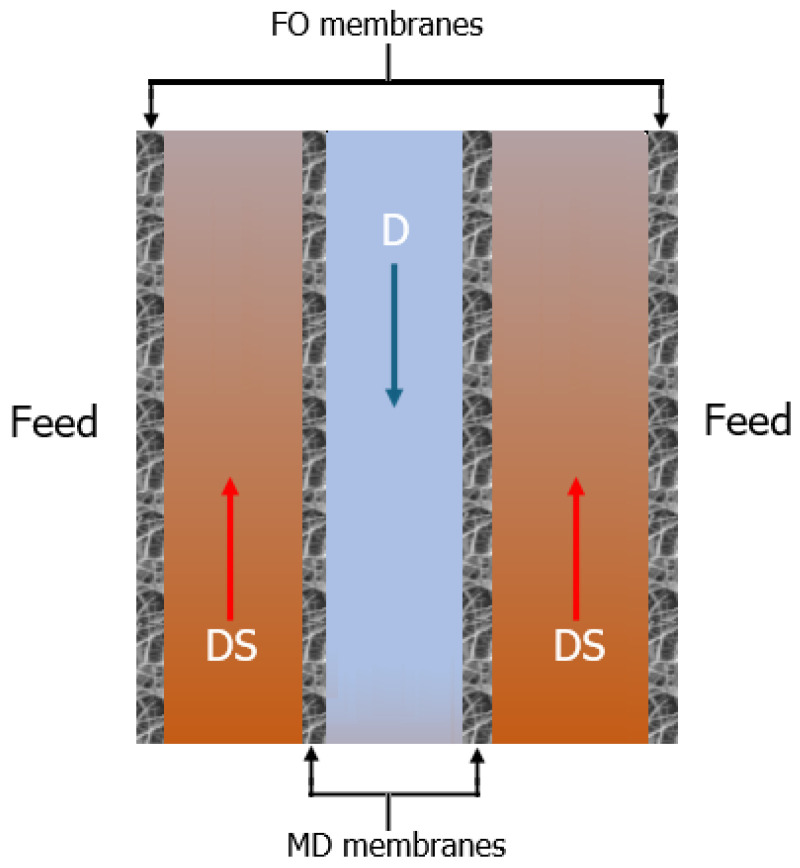
Schematic of an FO-MD module submerged in a feed solution (adapted from [211]).

Alternatively, an innovative FO-MD module, submerged in the feed solution and utilizing a plate-and-frame arrangement, has been assessed in the literature [211,212,213]. The module consists of three compartments, with the two FO membranes mounted on the external faces and two MD membranes mounted on the internal faces, as illustrated in Figure 11, which also indicates the flow directions. This FO-MD module reveals lower fluxes due to significant polarization effects, but it is less energy-intensive since it is not necessary to circulate the feed solution.

**Figure 12:**
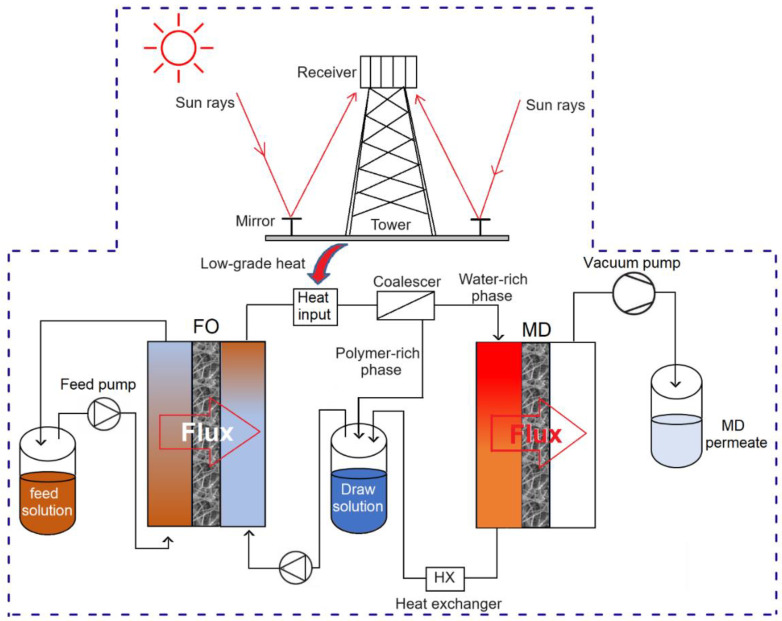
Integration of FO and MD in a hybrid process via a coalescer together with recovered waste heat from a concentrated solar power cycle.

Moreover, using thermo-responsive polymers as the draw solute offers the opportunity to integrate FO with MD via a coalescer. The diluted DS can be partially reconcentrated using the coalescer, followed by MD to produce high-quality water as the permeate and remove any residual polymer. In the DESOLINATION project [214], a thermo-responsive polymer was considered as the DS for the FO-MD hybrid process, as illustrated in Figure 12. When the diluted DS is heated through low-grade heat recovered from a concentrated solar power cycle, the polymer becomes less soluble in water and is partly separated from water using a coalescer. The water-rich phase from the coalescer is further purified by the MD unit, yielding high-quality water. The key advantage of a stimuli-responsive draw solute over conventional ones is the pre-separation of water from the DS performed by an external stimulus, leaving behind a far less concentrated stream for the secondary process. However, the associated high synthesis cost compared to conventional draw solutes like inorganic salts is the drawback of the stimuli-responsive draw solutes [25].

The combination of waste-heat availability and a high-osmolality draw solute is particularly well-suited for FO-MD hybrid processes, since the MD process is not substantially affected by the solute concentration [215]. This allows for efficient and stable feed water recovery when using MD [47]. Zhang et al. [123] proposed an FO-MD hybrid process for oily wastewater comprising surfactants, petroleum, NaCl, and acetic acid. A NaCl DS with concentrations varying from 0.58 to 5 M was used and achieved 90% water recovery while completely rejecting oil and NaCl. The low fouling propensity together with a high feed recovery ratio are the key benefits of this FO-MD hybrid process [47]. Lu et al. [216] utilized the thermal and osmotic energies of oily water in an FO-MD hybrid process to treat sewage, resulting in the recovery of ultra-pure water at a low-energy cost. The sewage was utilized as FO feed, while the oily water served as the MD feed and FO draw simultaneously. It was found that with the increase in oil content from 5 to 50 mg/L in the DS, and maintaining a salt concentration of 0.25 mol/L, the flux decline rate was less than 20% for both FO and MD.

The application of FO-MD at a large scale is limited owing to the high thermal energy needed for MD. Consequently, the need for residual heat is imperative to fulfill the thermal energy requirement. The potential to use low-grade energy sources is generally observed as a positive trade for MD, however, one of the obstacles faced by FO-MD pilots when combined with waste heat is integrating heat recovery and utilization into the system. Secondly, apart from the use of a low-grade heat source, module redesign and efficient internal heat recovery for MD remains a bottleneck for the technology to scale-up [217,218]. Another bottleneck that hinders FO-MD hybrid processes from large-scale commercialization is the high capital cost due to the individual MD and FO modules required. Therefore, the integration of FO with MD in one module, as discussed above, needs to be further investigated and employed on a large scale to reduce the capital cost of hybrid technology.

### 4.3. Fouling and Its Mitigation Through Advanced Membrane Material

As with all membrane systems, assessing and managing fouling in FO-MD hybrid processes is crucial due to its impact on operational efficiency, membrane longevity, and operating expenses arising from cleaning requirements [219]. In the FO-MD hybrid processes, the upstream FO process serves as the initial filtration stage, significantly reducing foulants and ensuring that the downstream MD process experiences minimal fouling and pore wetting. For instance, Xie et al. [95] demonstrated that treating raw sewage with an FO-MD hybrid process resulted in stable MD water flux, whereas standalone MD faced notable flux decline when raw sewage was fed directly. Similarly, another study [91] highlighted that feeding anaerobically digested sludge directly into the MD caused an 80% reduction in MD flux due to severe organic fouling. Compared to FO, MD is more susceptible to scaling, which diminishes membrane permeability and compromises water quality [191].

In the context of integrated FO-MD processes, several factors influence the formation of a fouling layer. One variable is the composition of the FS. For example, streams with high concentrations of organic pollutants often result in the development of organic fouling layers [220]. Conversely, feeds containing high inorganic content are more likely to clog the membrane pores [221]. Another critical factor is the type of membrane module used. Among the various designs, hollow fiber modules are most susceptible to fouling, followed by spiral wound, plate, and tubular modules [219].

In wastewater treatments, silica deposition is the most common type of fouling observed in FO systems due to its prevalence in water resources and its relatively low solubility [222,223]. Alongside silica, other compounds often cited in the literature as contributors to scaling in FO systems include gypsum, calcium carbonate, and calcium sulfate [224,225,226]. Compared with pressure-driven RO systems, fouling in FO membranes tends to occur more gradually and is generally reversible. Most organic and inorganic fouling agents can be effectively removed through backwashing or rinsing with tap water [227]. However, when high-concentration inorganic salts are used as draw solutes in hybrid FO-MD systems, they can lead to precipitation and deposition on the surfaces of MD membranes [228].

Besides the DS, the performance of the FO process depends on the type of membrane employed. Cellulose triacetate (CTA) and TFC membranes are mostly used for the FO process. CTA membranes are made from CA, whereas a typical TFC membrane comprises a thin active layer (10–200 nm thick) over a porous support layer (100–500 µm) [229]. The selective layer determines the FO flux, rejection, and RSF, whereas the porous layer provides support and controls the ICP and effective osmotic potential across the membrane [230]. In the commercial asymmetric FO membranes, two types of CP take place, i.e., ICP that occurs within the support layer, and external concentration polarization (ECP) that occurs on the membrane active layer. These ICP and ECP can either be dilutive or concentrative in nature depending on the orientation of the asymmetric FO membrane [231]. The presence of ICP in the support layer makes the FO membrane performance worse. The active layer of the asymmetric membrane facing the feed side is the preferred membrane orientation [222].

In recent years, advancement has been made in FO membranes by incorporating nanoparticles into the membrane structure to improve FO performance. Graphene oxide, zeolite, titanium dioxide, silicon oxide, and zinc oxide are some of the nanoparticles used in the literature to improve FO membrane performance and reduce fouling [232,233,234,235]. Li et al. [236] modified a TFC FO membrane by incorporating graphene oxide nanoparticles in the polyamide active layer and substrate to improve the water flux and anti-biofouling ability. They discovered that the membrane with graphene oxide in the substrate greatly improved the water flux due to improvements in the porous structure, whereas the membrane in which both the substrate and active layer were functionalized with graphene oxide showed higher anti-biofouling performance after 72 h of operation. Similar, Zirehpour et al. [237] functionalized the TFC FO membrane through nano-sized silver particles to improve the hydrophilicity of the active layer. The anti-fouling performance was tested and compared with the unmodified TFC membrane. The results indicated that during 1 day of operation, the water flux decline was about 7% for the modified membrane against a 18% decline for the unmodified membrane. A custom-made CA FO membrane has also been reported in an integrated FO-MD system [99]. These membranes are better than CTA in terms of flux, however, they have poor salt rejection performance [191,238].

In MD, polyvinylidene fluoride (PVDF), polytetrafluoroethylene (PTFE), and PP are the common polymers used for membrane fabrication [239]. The MD membrane should be hydrophobic and in particular, the characteristics needed for a MD membrane are a high LEP, high permeability, low fouling propensity, high chemical stability, lower material conductivity, and higher thermal stability [240]. Electrospinning [241] and the phase inversion method [242] are the common types of synthesis used for MD membranes.

To reduce the fouling propensity of hydrophobic MD membranes, common modification methods include graft polymerization, interfacial polymerization, and dip coating [243]. Nthunya et al. [244] coated a superhydrophobic PVDF membrane with an anti-fouling hydrophilic layer. The layer consists of silver nanoparticles and carboxylated multi-walled carbon nanotubes. Their DCMD investigations with bovine serum albumin (BSA) as the FS revealed a lower flux decline after long periods of operation compared to a pristine PVDF membrane. Zuo et al. [245] used a novel approach of grafting polyethylene glycol (PEG) onto the MD membrane surface for modification of PVDF membranes. The purpose of surface modification was to mitigate oil fouling. The modified membrane obtained a hydrophilic surface used for feeds containing oil. The novel membrane results in a stable MD flux of 6.3 LMH over 24 h of operation.

### 4.4. Applications of FO-MD Hybrid Processes

The applications of FO-MD hybrid processes range from complex waste streams like textile wastewater, landfill site leachate, and digested sludge to food applications. The goal in most applications is to achieve a highly concentrated FO retentate stream and purified water stream as MD permeate. Some key examples of FO-MD hybrid process applications are given as follows.

Textile and dying industry wastewater contains dyes, chemicals, and contaminants which are often difficult to treat. Ge et al. [99] employed an FO-MD process to recover wastewater and dehydrated acid dye from a dye industry, using PAA-Na as the draw solute. CA hollow fiber and PVDF composite flat-sheet membranes were used for the FO and MD processes, respectively. The optimized scheme developed indicates that efficient dehydration of the acid dye could be achieved at a draw solute concentration of 0.48 gm/L and a feed temperature of 66 °C for the MD unit. Similarly, Li et al. [246] demonstrated an FO-MD process for the reclamation of dye wastewater from the textile industry, achieving a concentration of 10 times the initial concentration.

**Figure 13:**
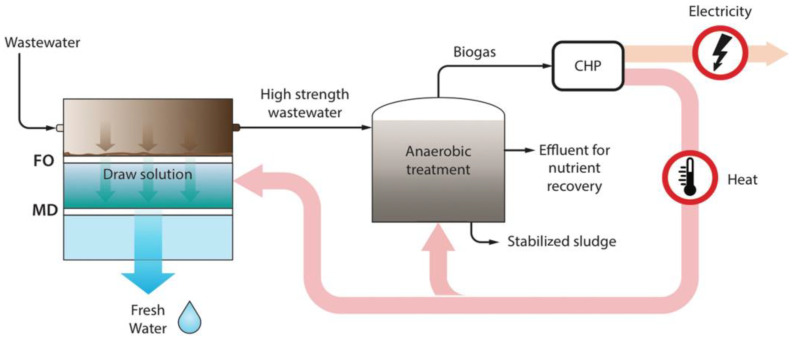
FO-MD hybrid process combined with an anaerobic wastewater treatment process (adopted from [251]). CHP stands for combined heat and power engine.

Furthermore, FO-MD hybrid processes can be also effectively used to treat digested sludge, removing nutrients such as nitrogen, potassium, and phosphorus, while producing high-purity water [247]. Husnain et al. [248] used the FO-MD process to treat nitrogen-rich feeds resulting from anaerobic digestion, successfully removing 98% of ammonia nitrogen and recovering 84% of the initial FO flux after a simple cleaning with tap water. A 1 M NaCl solution was employed as the DS. The high flux recovery was attributed to the reversible fouling characteristics of the FO process. Additionally, Xie et al. [91] demonstrated the use of the FO-MD for phosphorus removal from anaerobically digested sludge. In this case, FO removed phosphorus in the form of purified struvite, whereas 1.5 M MgCl_2_ DS was reclaimed through MD. When the sludge was fed directly into the MD, an 80% decrease in the MD flux was detected due to severe irreversible fouling. However, the FO membrane showed an 82% recovery of the initial flux with a single flush using DI water. This high recovery of the FO flux was attributed to the reversible nature of fouling in FO [249,250]. Ansari et al. [251] proposed an FO-MD hybrid process with an anaerobic membrane bioreactor to produce high purity water, as shown in Figure 13. The biogas produced from the anaerobic treatment process powers the combined cycle, generating both heat and electricity. This heat can be harnessed for the MD process, making MD the preferred option for DS regeneration.

FO-MD hybrid processes have also been successfully tested for high concentration sludge treatment. Several studies highlight the use of this hybrid process for concentrating nutrient-rich sludge, utilizing FO’s capability to enrich nutrients from concentrated streams [91,95]. Nguyen et al. [252] investigated the dewatering of the nutrient-rich sludge from domestic wastewater. Commercial TFC FO membranes from HTI, USA, and PTFE MD membranes from Ray-E Creative Co., Ltd., Taiwan, with three different pore sizes (0.1, 0.45, and 1 µm) were considered, while sodium phosphate was used as the DS and concurrently regenerated using MD. A concentration factor of 6.3 times the initial concentration was achieved after 15 h of operation.

Similarly, the FO-MD hybrid process was tested for the purification of landfill site leachate. Zhou et al. [96] optimized a hybrid process via response surface methodology with the flow rates and concentrations as key optimizing parameters. A commercial flat-sheet TFC membrane from HTI, USA, and microporous PTFE-PVDF composite membrane from Ming Lie Chemical Technology, China, were used in the FO and MD processes, respectively. The study revealed that with NaCl as the DS, the FO-MD process outperformed the standalone processes, achieving a salt rejection rate exceeding 96%, and TOC and total nitrogen (TN) rejections surpassing 98%. Additionally, the hybrid process fully rejected contaminants such as NH_4_^+^-N, Hg, Sb, and As. Similarly, Zhang et al. [228] examined the use of landfill leachate as the FS to study fouling control in an FO-MD process with 4 M NaCl as the DS. The findings showed a rejection rate of over 99% for the contaminants present in the FS, demonstrating the potential of the hybrid process for landfill leachate treatment. Pretreatment methods, such as electrocoagulation, can further improve the performance of this process [253].

The food industry has great potential for integrating FO-MD hybrid processes [254]. A limiting factor is currently the RSF, which must be either eliminated or managed by using food-compatible draw solutes [255]. An et al. [256] conducted a laboratory-scale study combining FO with MD to concentrate apple juice, utilizing potassium sorbate as the DS. A TFC FO membrane with a polyamide active layer was employed for FO, whereas a PVDF–hexafluoropropylene nanofibrous membrane was used in the MD process. Since potassium sorbate is commonly used as a food preservative, it served as a suitable DS for this application. The integrated system achieved apple juice concentrations of 4.25 times its initial concentration, with no loss of nutritional content, while simultaneously producing distilled water as the MD permeate. In a similar study, Song et al. [257] explored a laboratory-scale FO-MD hybrid process for dairy wastewater recycling with 1 M NaCl solution as the DS and employed MD for the DS regeneration. The study demonstrated a contaminant rejection rate exceeding 95%, with rejection rates for total phosphorus and TOC of over 99% and 96%, respectively.

Related to the life science and food industry is the use of FO-MD hybrid processes for protein enrichment, by combining the high-quality water yielded with high concentrations of proteins. Wang et al. [72] optimized the integrated process for BSA concentration by varying NaCl concentrations as well as MD feed temperatures. It was revealed that the FO-MD hybrid process remains stable when the FO permeation rate is equal to the MD permeate. Over a 4 h operation period, the BSA concentration increased from 1 g/L to approximately 2.1 g/L. However, to establish FO-MD hybrid processes for continuous protein concentration, it is crucial to minimize RSF into the feed stream [72,258].

Finally, beyond terrestrial applications, FO-MD has been explored for wastewater reclamation in space. Cath et al. [259] explored the hybrid process for space-based wastewater reclamation and urea removal. A CTA semi-permeable membrane from Osmotek Inc. and PTFE composite membrane from GE Osmonics were used in the FO and MD processes, respectively. A significant increase in flux (4–20 times) and 100% urea rejection compared to standalone FO was observed. In a related study, the FO-MD was used for the combined treatment of urine and humidity condensate [260]. As a post-treatment for MD permeate, catalytic oxidation was applied. The study confirmed that TDS, urea, and ammonia rejections in the process were more than 99.9%. FO-MD has also been considered as an applicable process for extracting urea and nutrients from human urine due to its high feed concentration capability [258,261,262]. Table 5 summarizes additional research conducted using the FO-MD hybrid processes.

### 4.5. Economics of FO-MD Hybrid Processes

Evaluating the sustainability of separation technologies, it is important to consider their economic viability. The FO-MD hybrid process provides dual barriers in the form of membranes, leading to higher associated costs compared to standalone NF/RO or FO/MD processes. The total cost of the technology includes both operational (OPEX) and capital expenditure (CAPEX).

**Figure 14:**
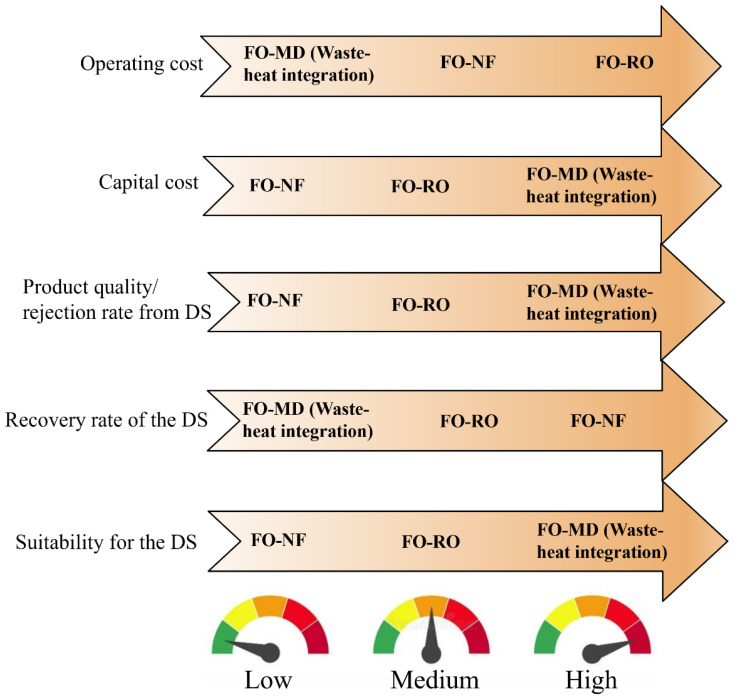
Comparison of secondary membrane processes used for DS regeneration [84,200,267,271,276,277,278,279] (Adopted from [191]).

CAPEX represents a major component of the overall cost of FO-MD, due to its technological complexity. CAPEX includes expenses such as membrane modules, pumps, pipes, fittings, electric cabinets, and measuring instrumentation [267]. The dual-membrane barrier concept inherent to hybrid processes typically results in higher membrane costs compared to standalone processes. OPEX encompass energy costs, membrane cleaning, labor costs, equipment maintenance, and membrane replacement [271]. Energy consumption accounts for a major portion of OPEX. Although FO is characterized by low energy consumption [272], the MD process, which is responsible for continuous DS regeneration and pure water production, contributes to an increased overall energy demand of FO-MD hybrid processes [273]. To reduce OPEX, renewable sources like geothermal, solar, or recovered industrial heat could be used to meet the high thermal demands of MD, potentially resulting in more competitive water production costs [11,274]. Cabrera-Castillo et al. [267] conducted an economic assessment comparing the water production cost of FO-MD and FO-RO hybrid processes, considering both conventional energy and waste heat sources. The study, using mining wastewater as feed, found that the FO-RO hybrid process incurred water production costs of USD 1.47/m^3^. In comparison, the FO-MD hybrid process with conventional heat sources had a cost of USD 3.36/m^3^, whereas the FO-MD hybrid processes driven by waste energy achieved a lower cost of USD 1.28/m^3^, outperforming the FO-RO hybrid process. This indicates that FO-MD hybrid processes can benefit from alternative energy sources, leading to a significant reduction in OPEX. The impact of thermal energy costs of MD on the overall OPEX of the FO-MD process is highlighted in a techno-economic study focusing on a standalone MD system. The water production costs with and without the integration of waste heat were evaluated to be about USD 0.74/m^3^ and USD 5.70/m^3^, respectively [271,275]. Furthermore, minimizing RSF to reduce the frequency of DS replenishment is another effective strategy for lowering the OPEX of the FO-MD hybrid process [267]. Therefore, careful selection of the DS and FO membranes to minimize RSF can further contribute to reducing OPEX. The comparison between common secondary processes used for DS regeneration are illustrated in Figure 14. The comparison was made considering the operating cost, capital cost, and rejection and recovery rates of the DS, and the suitability of the process for DS regeneration. The FO-MD process was evaluated with the integration of a waste-heat source as part of the system. When assessing the process based on the suitability of the DS, product quality, and operating costs, the MD process as a secondary step emerges as the most effective, suitable for both divalent and monovalent ionic solutions and having a higher rejection rate. On the other hand, if the comparison is based on capital cost and recovery rate of the DS, the NF process is superior.

A comparison of the OPEX for the membrane processes used for DS regeneration is presented in Table 6. Generally, the OPEX per unit produced decreases as the scale increases. With conventional heat sources for the MD, the OPEX follows the trend MD > ED > RO. However, when an alternative heat source is employed, large-scale MD becomes more cost-effective than RO and ED and the OPEX follows the trend ED > RO > MD.

Higher fluxes in the FO-MD can lower the CAPEX by lowering the required membrane area needed to get the same permeate flow rate [83]. Zarebska-Mølgaard et al. [287] performed a techno-economic estimation of the FO-MD process, revealing water production costs of 1.29 USD/m^3^ and 2.96 USD/m^3^ considering FO and MD fluxes of 10 and 2 LMH, respectively. Their findings indicated that the FO-MD hybrid process becomes economically viable as the combined water flux exceeds 14 LMH [287]. Additionally, extending the membrane life cycle is another key factor for reducing OPEX [288]. In the FO-MD process, the FO stage effectively removes most contaminants, thereby reducing the likelihood of MD membrane wetting and thus extending the MD membrane life cycle [206,289].

The replacement of the FO membrane represents approximately 60.8–86.2% of the OPEX associated with the FO stage [246]. Due to the lower fluxes of FO membranes, a larger membrane area is required to achieve equivalent flux to that of MD, leading to increased FO membrane costs. For instance, Yangali-Quintanilla et al. [290] reported the cost of FO membranes to be 30 and 60 USD/m^2^. MD membrane costs are reported in the same price range of 36 USD/m^2^ [280] and 60 USD/m^2^ [291] for commercial MD membranes, leading to approximately 4% of the OPEX associated with the MD stage. In the FO process, membrane fouling is often reversible, and most organic and inorganic foulants can be removed through backwashing or rinsing with tap water [227]. Backwashing can restore over 90% of the FO membrane’s capacity, significantly reducing the need for chemical agents and lowering the OPEX [48,49]. This was further demonstrated by Li et al. [246], who reported cleaning costs of 0.030, 0.025, and 0.024 USD/m^3^ of permeate for custom-made symmetric membranes, commercial CTA membranes from HTI, USA, and commercial TFC FO membranes from Toray Chemical, South Korea, respectively. Overall, cleaning costs for FO membranes are approximately 30% lower compared to those for RO membranes [83].

Fouling control and energy savings enable FO-based hybrid processes to achieve OPEX that are approximately half those of conventional RO [83]. However, MD highly relies on thermal energy, which can lead to elevated OPEX and water production costs in comparison to other hybrid processes like FO-RO hybrid processes, if alternative or waste-heat resources are not utilized [267]. Therefore, the integration of MD with some form of renewable energy source or waste heat significantly reduces its OPEX [267,292,293,294]. Al-Obaidani et al. [11] found that integrating MD with conventional or low-grade heat yielded water production costs of 1.23 and 0.64 USD/m^3^, respectively. Likewise, Kesieme et al. [283] and Hanemaijer et al. [295] reported water production costs of 0.66 and 0.26 USD/m^3^, respectively, when utilizing industrial waste heat, underscoring the potential of the MD process for efficient use of low-grade heat. Despite the significant potential for utilizing industrial waste heat to drive thermal desalination processes like MD, two factors must be considered [296]. The first is the additional CAPEX associated with the installation of the waste heat recovery unit, which increases the product water desalination cost in certain cases. The second is the heat recovery efficiency, which varies depending on the waste heat source, the recovery approach, and the recovery system used. In the case of the FO-MD hybrid process, a heat-to-heat recovery approach, such as a heat exchanger, can effectively transfer heat from waste heat to useful heat.

### 4.6. Energy Consumption in FO-MD Hybrid Processes

In the FO step of the FO-MD process, electricity is the primary energy source, mainly consumed for pumping the DS and FS [48]. Although less frequently reported, energy consumption related to the pretreatment of the FS may also be significant [297]. The pumping energy can be calculated as follows [298]:

(3) $${E\left(FO\right)}_{pumping}=\frac{m^{.}\Delta P}{\eta_{p}}$$

where $m^{\cdot }$ represents the mass flow rate, ΔP is the pressure drop, $\eta_{p}$ denotes the pump efficiency, and ${E\left(FO\right)}_{pumping}$ is the pumping energy needed for the FO process. To minimize energy consumption in FO, it is crucial to enhance the pumping efficiency. Additionally, since pumping energy depends on the mass flow rate, use of variable frequency drive pumps is recommended to further reduce its consumption [191]. The electrical energy required for other components, such as sensors, is almost negligible.

In MD, Equation (3) is also applicable to calculate the pumping energy required. However, the primary cost consideration is the thermal energy consumption, as MD relies on the latent heat of evaporation to vaporize the permeate [299]. Experimental analysis by Li et al. [246] demonstrated that the specific energy consumption (SEC) for MD was only 7.2–17.5% higher than the STEC, indicating a strong reliance on thermal energy. The required thermal energy for MD is provided by the following equation:

(4) $${E\left(MD\right)}_{thermal}=m\cdot \Delta H=m\cdot C_{p}\left(T_{f,in}-T_{f,out}\right)$$

where ΔH is the change in the enthalpy of the FS upon heating, $C_{p}$ represents the specific heat capacity, and $T_{f,in},\;T_{f,out}$ corresponds to the temperatures at the feed inlet and outlet, respectively. This equation illustrates that the thermal energy demand is directly related to the temperature difference between the feed inlet and outlet. MD is characterized by three types of heat transfer: convection, conduction, and latent heat loss associated with the vapor transport across the membrane. Additionally, TP in MD can lead to a higher thermal energy requirement compared to the theoretical estimate. Strategies to mitigate the high demand for thermal energy include reducing the TP effect by incorporating turbulence promoters, opting for membrane materials with lower thermal conductivity (e.g., polymeric membranes), incorporating multi-staging in MD configurations, and employing photothermal heating [172,174,183,300].

The SEC of the FO-MD hybrid process can be determined as follows:

(5) $${SEC}_{FO-MD}=\frac{{E\left(FO\right)}_{pumping}+{E\left(MD\right)}_{pumping}+{E\left(MD\right)}_{thermal}}{{WPR}_{MD}}$$

where ${WPR}_{MD}$ is the water production rate and is calculated as follows:

(6) $${WPR}_{MD}={Area}_{MD}\cdot {Flux}_{MD}$$

The SEC is significantly influenced by the permeate flux and membrane area in both FO and MD. As indicated by Equations (5) and (6), increasing either the permeate flux or effective membrane area results in a reduction in the SEC, regardless of the membrane type employed [246]. However, the overall SEC of the FO-MD hybrid process tends to be higher, particularly when a conventional heat source is utilized for the MD step. Giagnorio et al. [301] reported that the overall SEC required to drive an FO-MD hybrid process is approximately twice that of an FO-RO hybrid process.

Energy consumption in the FO step is relatively low. Mazlan et al. [22] estimated the SEC for the FO to be as low as 0.11 kWh/m^3^ at a 50% recovery rate. However, the integration of MD for DS recovery negates the low SEC benefits achieved by the FO stage. Dow et al. [205] conducted a pilot-scale DCMD study utilizing waste heat from a textile factory to treat textile wastewater from Australian Textile Mills, revealing an average STEC of 1600 kWh/m^3^ of clean water permeated. The STEC increased significantly, reaching 4180 kWh/m^3^ prior to the cleaning shutdown, which underscores the significant thermal energy requirements of the MD process.

Waste heat utilization has significantly reduced desalination costs and environmental impacts for thermal energy-based desalination processes, as shown in Table 6 [302]. Approximately 20–50% of the energy utilized in the US industry is lost as waste heat in the form of hot exhaust gases annually [303,304]. A report from the US Department of Energy has showed that out of the 2462 TWh/yr energy utilized in the industrial sector, 440 TWh was lost as waste heat [278,305]. Similarly, another study estimated the total residual heat to be 163 million GJ/year from all the exhaust streams across the United States [306]. Thus, this waste heat could be used as a sustainable heat source to power the MD process.

To confirm whether the waste heat from an industry would be adequate to run an FO-MD process, Anderson et al. [307] applied a bench-scale FO-MD setup to treat wastewater produced at a coal-fired power plant and performed heat analysis of the plant. By using three different DSs (NaCl, CaCl_2_, and PAA-Na), the FO-MD performance was assessed across a range of MD feed temperatures (43.5–65.5 °C). The FO-MD setup achieved 99.8% rejection of the components from wastewater and produced distillates with conductivities lower than 105 µS·cm^−1^. The 1300 MW_ele_ power plant required treatment of the generated wastewater at a rate of 1500 LPM. The heat source in the power plant was the cooling water flowing at 2,270,000 LPM, with a temperature range of 27–43 °C. Based on the temperature and flow rate of the cooling water, the available thermal energy that could be harvested by MD ranges from 1000 to 3500 MW_th_. The following equation is used to determine the thermal energy demand of the FO-MD process.

(7) Thermal energy=STEC·Flowrate of the waste water

A thermal energy of 280 MW was required to clean 1500 LPM of wastewater from the power plant. Thus, the residual heat available in the cooling water was 3.5 to 12.5 times that needed to meet the thermal energy demands.

### 4.7. Scale-Up of the FO-MD Hybrid Processes

The scale-up of the FO-MD hybrid processes necessitates addressing several key challenges, which require consideration of multiple aspects. Firstly, the advancement of effective membrane materials for FO remains limited, which hampers large-scale applications of FO [65,308,309]. Incorporating nanoparticles could improve the FO membrane properties, however, this has inherent difficulties in terms of cost and large-scale production [310]. Additionally, the commercialization of FO is limited by the need for a DS which exhibits both high osmotic pressure and minimal RSF.

Energy consumption is generally a key aspect in the sustainability and scalability of membrane processes. The high thermal energy demands of MD pose a significant hurdle to its commercialization. To enhance the viability of MD for large-scale applications, it is essential to explore alternative energy sources, like waste heat recovered from industrial processes or power plants [311,312]. Thus, advancing FO-MD hybrid processes requires focused research on two primary areas: development of commercially viable FO membranes and optimization of the MD stage to reduce energy consumption.

**Figure 15:**
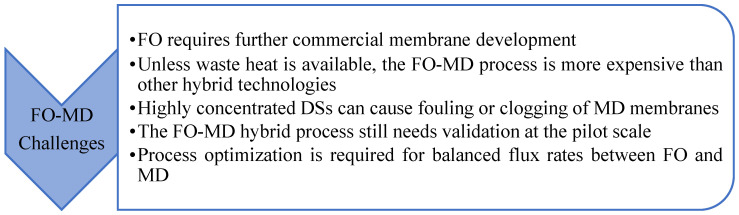
Limitations/challenges of FO-MD hybrid processes.

The integration of FO with MD in a hybrid process provides several synergistic advantages that mitigate the limitations inherent in each individual technology [313,314]. Despite the robustness and potential for various applications, research on FO-MD hybrid processes is predominantly limited to laboratory-scale studies [315]. To unlock the full potential of the hybrid process, it is vital to scale up the process from laboratory to pilot-scale operations. Therefore, conducting a detailed techno-economic analysis of pilot-scale FO-MD hybrid processes is crucial to assess their viability as commercial technology. Furthermore, it is essential to address the various other limitations associated with the FO-MD process, as shown in Figure 15. This is essential as most challenges related to the scale-up of the FO-MD hybrid process are linked to economic aspects.

## 5. Conclusions and Outlook

This review article examines the limitations of standalone FO and MD processes and highlights the benefits of integrating them into a hybrid process. The key findings include the following:

- Despite progress in membrane technology, further research is essential in areas such as energy-efficient module design and scalable configurations to bring the technology closer to commercialization.
- An optimal DS must offer high osmotic pressure, a high diffusion coefficient, increased solubility, low molecular weight and viscosity, minimal RSF, cost-effective recovery, and compatibility with membranes and human consumption-related applications, such as food and drinking water. However, existing DSs are economically not feasible, due to either high costs or expensive regeneration processes.
- Although FO-MD hybrid processes demonstrate significant promise for industrial applications, particularly in treating complex wastewater, they remain so far predominantly at the laboratory scale due to the challenges associated with DS selection and its regeneration costs.
- The FO-MD hybrid process has the potential to achieve significantly lower OPEX compared to other membrane-based hybrid systems, such as FO-RO, particularly when utilizing waste or renewable energy sources for MD. For example, an FO-MD system powered by waste heat can produce water at a cost of USD 1.28/m^3^, making it 61.9% cheaper than using a conventional heat source for FO-MD, which costs USD 3.36/m^3^. Additionally, it is 12.93% more cost-effective than the FO-RO process, which produces water at USD 1.47/m^3^.
- Although there have been few studies examining FO-MD hybrid processes on an industrial scale, such as wastewater treatment utilizing waste heat, there is a pressing need for further techno-economic analysis to assess its feasibility in various industries.

Overall, the primary opportunity for the FO-MD hybrid process lies in identifying suitable DSs and utilizing renewable or low-grade energy sources, which are crucial for the technology’s successful upscaling and implementation.

## Figures and Tables

**Table 5:** Selected applications of FO-MD hybrid processes.

FS, DS, and Membranes	Conditions and Results
FS: raw sewage from a sewage treatment plant, DS: NaCl solution. FO membrane: commercial CTA flat-sheet membrane HTI (USA). MD membrane: PTFE membrane from porous membrane technology (Ningbo, China).	FO feed and draw CFV: 9 cm/s, FS temperature: 20 °C, DS temperature: 40 °C, DS concentration: 1.5 M NaCl, FO flux: 8 LMH. MD feed and distillate flow rates: 1 L/min, feed temperature: 40 °C, distillate temperature: 20 °C, MD flux: 8 LMH. Achieved 80% recovery of the raw sewage by the FO-MD hybrid system. A cumulative permeate volume of 8.25 L was attained after 78 h [95].
FS: DI water, municipal wastewater. DS: NaCl solution, TEAB, and PDAC. CTA membrane from HTI (USA).	FO CFV: 0.12 m/s, DS concentration: 0.5 M NaCl, 0.44 M PDAC, and 0.5 M TEAB. MD with three different feeds: 0.5 M NaCl, 0.44 M PDAC, and 0.5 M TEAB. Feed temperature: 45 °C, permeate temperature: 20 °C, CFV: 0.12 m/s. FO-MD flux: 6–8 LMH. More than 99% rejection of all DSs through MD [93].
FS: DI water, 0.3–0.495 g/L of NH_4_Cl solution, non-fat dry milk with concentration of 985–1800 mg/L COD, synthetic wastewater, real wastewater, and FS containing 0.1–0.2 g/L NaAsO_2_. DS: 1 M NaCl. FO membrane: CTA membrane HTI (USA).	FO feed temperature: 20 °C, DS temperature: 50 °C. MD feed temperature: 50 °C, permeate temperature: 20 °C, CFV: 0.4 L/min. Feed as DI water: 14.4 LMH of flux for both FO and MD. Feed with 300–495 mg/L of ammonium concentration, ammonium detected in the permeate was 0.18 mg/L, indicating 100% rejection. COD removal from non-fat dry milk was 90% by FO alone, preventing fouling and wetting in MD. More than 99.9% removal of the chemical solutes from synthetic and real wastewater [202].
FS: wastewater. DS: NaCl solution. FO membrane: commercial flat-sheet CTA membrane from HTI (USA). MD membrane: PVDF hollow fiber membrane.	FO DS concentration: 35 g/L, feed and DS CFV: 0.28 m/s. DS temperature: 53 °C, FS temperature: 20 °C. MD flux: 17.6 LMH, product water conductivity: 7.5 µS·cm^−1^. More than 90% removal efficiency of the contaminants (Cl^−^, NO_3_^−^, SO_4_^2−^, PO_4_^3−^, TOC, NH_3_-N, TN, and total phosphorus) was obtained [263].
FS: raw flue gas desulfurization wastewater. DS: 3 M NaCl. FO membrane: TFC polyamide membrane from Porifera, Inc. (San Leandro, CA, USA). MD membrane: PTFE supported by PP from Pall Gelman Sciences (Port Washington, NY, USA).	FO process CFV: 8.5 cm/s, temperature: 25 °C. FO process was carried out under the AL-FS mode. MD feed and permeate temperatures were 55 and 20 °C, respectively. At a 50% recovery rate, flux decreased from 18.5 to 13.1 LMH. Afterward, a sharp decrease in flux (13.1 to 2.8 LMH) was observed at 55% recovery. An organic removal rate of 98.61% was achieved [264].
FS: dairy wastewater. DS: 2 M NaCl. FO membrane: commercial flat-sheet CTA membrane from HTI (USA). MD membrane: PP and PVDF membranes from GE Osmonics (Minnetonka, MN, USA).	FO feed and DS flow rates: 300 L/h, temperature: 25 °C. Feed and DS CFV: 0.5 m/s. MD feed temperature: 48 °C, distillate temperature: 20 °C. Feed and permeate crossflow rates: 180 L/h, MD (PVDF) flux: 17.1 LMH, MD (PP) flux: 27.7 LMH [265].
FS: wastewater from the pharmaceutical industry. DS: 0.6 M NaCl. FO membrane: polyamide membrane. MD membrane: flat-sheet PVDF membrane.	FO feed as 500 mg/L tetracycline solution, feed and DS CFV: 12.5 cm/s, FS temperature: 25 °C, DS temperature: 70 °C, FO initial flux: 40 LMH. MD transmembrane temperature: 43 °C, feed CFV: 1.67 cm/s, MD flux: 18 LMH. Rejection of tetracycline by FO-MD: 99.9%. After 7 h run, tetracycline content in distillate water was less than 0.1 mg/L [266].
FS: acid mine drainage. DS: 1 M NaCl. FO membrane: flat-sheet TFC membrane from Porifera, Inc. (USA). MD membrane: PTFE/PP flat sheet from Membrane Solutions (Shanghai, China).	FO FS temperature: 20–22 °C, DS temperature: 60 °C, FO initial flux: 23 LMH. After 12 h, FO flux was 64% of the initial value. MD feed temperature: 60 °C, distillate temperature: 20 °C, flux: 10.5 LMH with only a small decrease during 3.5 h of operation. 50% recovery was achieved [267].
FS: oily wastewater. DS: 5 M NaCl, 4 M KCl, 4.8 and 10 M LiCl, 4.8 M MgCl_2_. FO membrane: commercial TFC membrane from HTI (USA). MD membrane: PP flat-sheet membrane obtained from 3 M^®^.	FO FS temperature: 20 °C, DS temperature: 50 °C, CFV: 0.25 m/s. MD feed temperature: 50 °C, permeate temp: 20 °C, CFV: 0.25 m/s. LiCl showed higher flux in FO since the osmotic pressure is more at higher concentrations. 10 M LiCl exhibited lower MD flux due to lower vapor pressure [268].
FS: oily wastewater. DS: 2 M NaCl. FO membrane: CTA-TFC membrane. MD membrane: PVDF hollow fiber.	FO FS temperature: 23 °C, DS temperature: 60 °C. FO flux: 20–32.5 LMH with oily wastewater (4000 ppm petroleum) as feed. MD feed temperature: 60 °C, MD flux: 5.8 LMH [123].
FS: synthetic oily water, sewage. DS: oily water (5–50 mg/L of oil concentration). FO membrane: CTA membrane HTI (USA).	FO DS temperature: 50 °C, DS concentration: 5–50 mg/L of oil concentration. FO flux: 0.65 LMH. FO-MD: at 50 mg/L of oil concentration in DS, flux declined from 5 to 2.6 LMH [216].
FS: shale gas drilling fluid from a drilling site. DS: KCl, NaCl, and MgCl_2_. FO membrane: commercial CTA membrane obtained from HTI (USA). MD membrane: CF_4_-plasma-modified PVDF membrane.	FO feed and DS flow rates: 0.6 L/min, DS concentration: 3 M KCl, 3.26 M NaCl, and 1.75 M MgCl_2_. Order of FO water flux at the same osmotic pressure: KCl > NaCl > MgCl_2_. MD process: VMD configuration. Applied vacuum: −40 kPa. Sweeping air flow rate: 6 L/min. 23 LMH of flux were achieved at 3 M KCl as DS at a temperature of 25 °C [269].
FS: wastewater. DS: 1.5 M Na_2_SO_4_. FO membrane: CTA membrane from HTI (USA). MD membrane: PTFE membrane from Sterlitech Corporation (Auburn, WA, USA).	FO feed and DS cross flow rates: 0.2 L/min, FS temperature: 25 °C. MD feed flow rate: 0.2 L/min, distillate flow rate: 0.1 L/min, feed temperature: 55 °C, distillate temperatures: 5, 15, and 25 °C. Optimal MD performance at a temperature difference of 50 °C. Flux: 18.6 LMH, RSF: 5.1–8 gMH [246].
FS: seawater. DS: NaCl 70,000 ppm. FO membrane: Spiral-wound TFC membrane from Toray (USA). MD membranes: Spiral-wound AGMD module from Aquastill (Sittard, The Netherlands).	FO feed and DS flow rates: 600 L/h and 400 L/h, respectively. FS temperature: 25 °C, flux: 6.3 to 7.3 LMH. MD process: AGMD configuration. Feed temperature: 85 °C, permeate temperature: 10 °C, flux: 3.75 to 4 LMH. Average water recovery: 33% [270].

TEAB—Tetraethyl ammonium bromide; PDAC—Polydiallyldimethylammonium chloride.

**Table 6:** OPEX estimation of the common membrane processes used for the DS regeneration.

Membrane Process	Capacity (m^3^/Day)	Conditions	Heat Source for MD	OPEX (USD/m^3^)	Ref.
MD	0.1–0.5	Brackish water and untreated seawater as feed, spiral air gap MD modules.	Solar energy	15–18	[280]
MD	0.51	Four stage multi-effect air gap MD module. Membrane: commercial flat-sheet PTFE membrane (Madhu Chemicals, Mumbai, India).	Conventional heat source with heat recovery	8.91	[281]
MD	0.51	Four stage multi-effect air gap MD module. Membrane: commercial flat-sheet PTFE membrane (Madhu Chemicals, India).	Waste-heat source	3.82	[281]
MD	24,000	DCMD configuration.	Conventional heat source without heat recovery	1.23	[11]
MD	24,000	DCMD configuration. Feed inlet temperature: 55 °C.	Industrial waste heat without heat recovery	0.64	[11]
RO	1000	-	-	0.82	[1,282]
MD	30,000	DCMD configuration, flat-sheet PTFE membrane from Ningbo, China. Feed temperature: 60 °C.	Driven with high-temperature waste heat and electricity	0.66	[283]
ED	100	-	-	1.87	[284]
MD	30,000	DCMD configuration, flat-sheet PTFE membrane with PP support from Ningbo, China.	Driven with low-temperature waste heat and electricity	0.57	[283]
RO	275,000	-	-	0.5	[285]
MD	105,000	Memstill^®^ technology with hollow fiber membranes, feed inlet temperature: 65 °C.	Industrial waste heat source at USD 0.10/GJ	0.26	[148]
RO	2.6	-	-	3.61	[286]
